# Abstract and concrete concepts in conversation

**DOI:** 10.1038/s41598-022-20785-5

**Published:** 2022-10-20

**Authors:** Caterina Villani, Matteo Orsoni, Luisa Lugli, Mariagrazia Benassi, Anna M. Borghi

**Affiliations:** 1 Department of Philosophy and Communication, University of Bologna, Via Azzo Gardino, 23, 40122 Bologna, Italy; 2 Department of Psychology, University of Bologna, Bologna, Italy; 3 Department of Dynamic and Clinical Psychology, and Health Studies, Sapienza University of Rome, Rome, Italy; 4 Institute of Cognitive Sciences and Technologies, Italian National Research Council, Rome, Italy

**Keywords:** Psychology, Human behaviour

## Abstract

Concepts allow us to make sense of the world. Most evidence on their acquisition and representation comes from studies of single decontextualized words and focuses on the opposition between concrete and abstract concepts (e.g., “bottle” vs. “truth”). A significant step forward in research on concepts consists in investigating them in online interaction during their use. Our study examines linguistic exchanges analyzing the differences between sub-kinds of concepts. Participants were submitted to an online task in which they had to simulate a conversational exchange by responding to sentences involving sub-kinds of concrete (tools, animals, food) and abstract concepts (PS, philosophical-spiritual; EMSS, emotional-social, PSTQ, physical-spatio-temporal-quantitative). We found differences in content: foods evoked interoception; tools and animals elicited materials, spatial, auditive features, confirming their sensorimotor grounding. PS and EMSS yielded inner experiences (e.g., emotions, cognitive states, introspections) and opposed PSTQ, tied to visual properties and concrete agency. More crucially, the various concepts elicited different interactional dynamics: more abstract concepts generated higher uncertainty and more interactive exchanges than concrete ones. Investigating concepts in situated interactions opens new possibilities for studying conceptual knowledge and its pragmatic and social aspects.

## Introduction

Concepts allow categorizing objects and entities, making inferences based on previous experiences, and preparing to act^1^. Many have distinguished concepts into concrete and abstract concepts (from now, CCs and ACs) (e.g., “table” vs. “justice”, but also “stop” and “maybe”^2^). Recent views see CCs and ACs as neither dichotomously opposed nor as representing a continuum. Instead, different concepts would be points within a multidimensional space, defined by various dimensions^3^. Studies have identified some of these dimensions, none of which is exhaustive or necessary. Compared with more CCs, ACs generally include more heterogeneous members (density^4^); they are more detached from the five senses, less imageable^5^, and evoke more frequently inner experiences (interoception, emotions)^6,7^. Furthermore, they refer to relations rather than single objects^8^, are less iconic^2^, and more variable across contexts^9,10^. The words expressing them are typically acquired later and through language rather than through perception^11^. Language and social interaction are crucial for their acquisition and representation^12–14^. Because of their complexity, ACs might lead to higher uncertainty, less confidence in their meaning, and stronger involvement of metacognition and inner speech^15^.

Importantly, some have recently acknowledged that ACs come in different kinds^16^, for which different dimensions are relevant^17^. Thus, emotional and aesthetic ACs evoke more interoception and emotions^18^, numerical ACs elicit more sensorimotor experiences linked to finger counting^19^. All ACs, just as CCs, activate sensorimotor brain areas. A meta-analysis demonstrated that numerical, emotional, morality, and theory of mind ACs also engage specific brain areas^20^. To date, converging evidence supports the existence of different types of ACs. They span in a multidimensional space that includes interoceptive, sensorimotor, affective, social, and linguistic features to a different extent (review^21^).

Understanding how ACs are represented constitutes a challenge for both embodied theories, according to which the body influences and constrains cognition, and distributional theories, according to which we get meaning through word associations. Hybrid, multiple representation views represent a promising alternative^22–24^. They relate the differences between CCs and ACs and their kinds to the different weights sensorimotor, interoceptive, linguistic, and social experiences play.

Crucially, the emergent ways to conceive concepts impose the adoption of new methods^25–27^. Most studies so far have focused on single words or simple sentences. Typical methods are ratings of different dimensions (imageability, contextual availability, Age of Acquisition, Modality of Acquisition, Emotionality)^28,29^, and feature listing and definitions^30,31^. Among the implicit tasks, the most common are lexical decision, recall, recognition, and property verification tasks (e.g.,^32–34^). Brain imaging studies typically use words or simple sentences differing in imageability or abstractness^23,35,36^. Conversely, studies on natural conversations^37^ rarely focus on ACs and CCs. In one of the few studies on interaction, participants had to explain the meaning of CCs and ACs, avoiding using the word themselves (taboo game) (^38^; see also^39^).

An effective approach for investigating conceptual representation is to infer it from the use of concepts in interaction. According to some recent theories, social interaction is crucial for abstract concepts. Because their referents are not objects and the members of abstract categories are very heterogeneous, other people are particularly crucial to help us acquire them. In addition, we have proposed elsewhere that other people might be particularly important also during abstract concepts processing. Through interaction, other people can facilitate our processing of abstract words, either helping us understand the meaning of the words or negotiating the word's meaning with us^40,41^. Because of the crucial role social interaction might play for abstract concepts, it becomes pivotal to investigate them through interactive tasks. The present work aims at exploring how abstract and concrete concepts are used in conversation. In our study, participants engaged in exchanges starting from different kinds of concepts. We presented three kinds of CCs, the most common in the literature (tools, animals, and food), and three kinds of ACs, derived from a previous rating study, i.e., philosophical-spiritual, PS (e.g., “value”), physical-spatio-temporal-quantitative, PSTQ (e.g., “mass”), emotional-mental states and social concepts, EMSS (e.g., “anger”)^17,28^. The previous norming study^28^ showed that PS concepts resulted as more abstract, EMSS characterized mostly by inner experiences, and PSTQ more based on sensorimotor experience than other ACs. In this study, we asked participants to simulate a conversational exchange with another person, responding to a written sentence focused on a concrete/abstract concept (e.g., I made a cake/a judgment). We then used the Interpersonal Reactivity Index (IRI^40^) questionnaire to explore whether individual differences in empathy influenced the responses, especially for those prompted by ACs that may rely heavily on emotions and affective states^7,32^. Since we hypothesize that, with abstract concepts, people rely more on others^41^ we intended to explore whether there is a relationship between conceptual abstractness and the level of empathy demonstrated by participants.

We hypothesized that responses to sentences involving ACs would differ from those involving CCs and intended to explore the dimensions distinguishing concept kinds. Notably, some of these dimensions allow inferring how concepts are represented from the conversational pattern they evoke. We outline below the hypotheses we pre-registered and tested; for each of the six hypotheses, we also explored eventual differences between kinds of ACs and CCs.

1. Conversation: Uncertainty expressions, Number of questions, and Target word repetitions. We expected (directional hypothesis) more uncertainty expressions (e.g., “mmm…”, “I am not sure” , “What do you mean?”) and signs of uncertainty, like questions and repetitions of the target word, with ACs than CCs (moderate to strong evidence).
2. Conversation: Turn-taking (directional hypothesis). With ACs participants should be more uncertain on the word meaning and need to rely on others more^15,41^; hence, with ACs, participants should more often continue the discussion assigning a further turn to the other either by asking questions or by using expression signaling the willingness of knowing more details (e.g., “Tell me more”, “Explain it to me”), thus eliciting a social interaction dynamic (moderate to strong evidence).
3. Conversation: Point of views and General Statements. We investigated whether the participants made a general statement (e.g., “Dream is important”, “Revenge is a human feeling”) or whether s/he considers the other’s perspective (e.g., “You were right”, “Don’t give up”). We referred to this with Points of View. Specifically, we distinguished between 1st, 2nd, and 3rd person Point of View and the coupling of 1st and 2nd person (i.e., interpersonal Point of view). We expected more general statements with ACs than CCs.
4. Number of evoked contexts. Because ACs meaning is more context-dependent^9^, we expected that participants would refer to more contexts with ACs than CCs (moderate to strong evidence).
5. Produced features. We expected (directional hypothesis) that (a) ACs are more focused on internal situational elements^12,25^, i.e., leading to the production of beliefs (e.g., “I think that…” “I should do that”, “It was necessary”), evaluative (not perceptual, e.g., “it is useful!”, “great”, “It was correct”), emotional, (e.g., “I am happy/glad/nervous”, “I hope it works”), introspective (e.g., “I remember when…”, “I often feel ashamed when I speak in public”), and metacognitive properties (e.g., “I am good at memorizing”, “I am not able to cook”); (b) CCs evoke more external sensorimotor and contextual properties, i.e., evaluative (perceptual) properties experienced through five senses and thematic spatial and temporal relations, particularly those related to action/agency (moderate to strong evidence). We also expected emotional concepts to activate more emotional and interoceptive features than other concepts.
6. Conversation: Kind of questions (how, why, where, what, when, who). We predicted more “why” questions with ACs, the meaning of which generates more uncertainty and more questions related to external situational elements (“what”, “where”, and “when” questions) with CCs (moderate to strong evidence).

## Results

**Figure 1:**
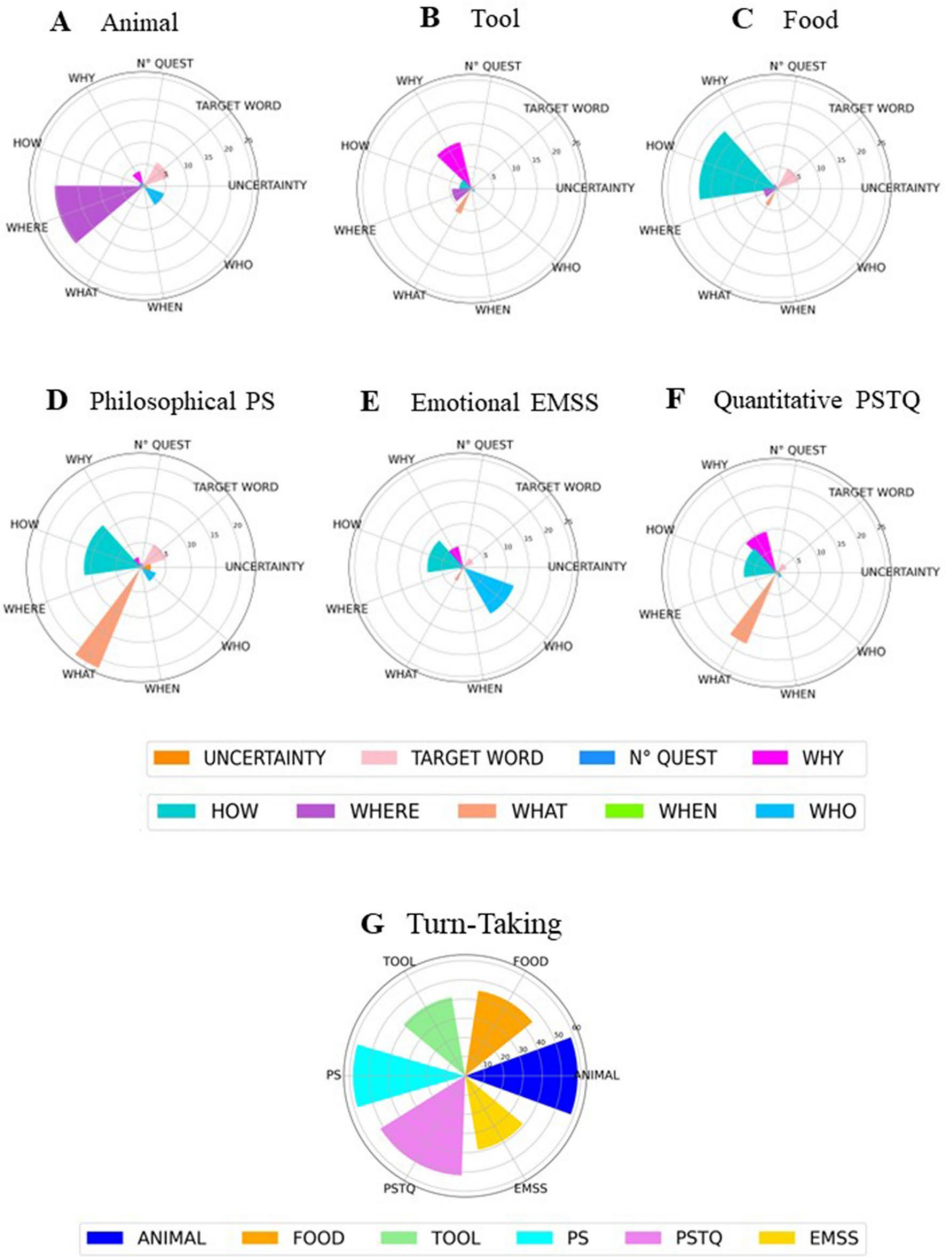
Polar plots for sentences including animals (**A**), tools (**B**), food (**C**), PS, philosophical-spiritual (**D**), EMSS, emotional-social (**E**), and PSTQ, physical-spatio-temporal-quantitative (**F**) concepts, showing the row frequency count of the variable number of questions (dodger blue), the percentage of the other coded variables concerning conversation i.e., uncertainty expressions (dark orange), target word repetition (pink), why (magenta), how (dark turquoise), where (medium orchid), who (deep sky blue), when (lawn green), and what (light salmon) questions. A polar plot showing the percentage of the turn-taking variable (**G**) for sentences including animals (blue), tools (light green), food (orange), PS, philosophical-spiritual (aqua), EMSS, emotional-social (gold), PSTQ, physical-spatio-temporal-quantitative (violet) concepts.

**Figure 2:**
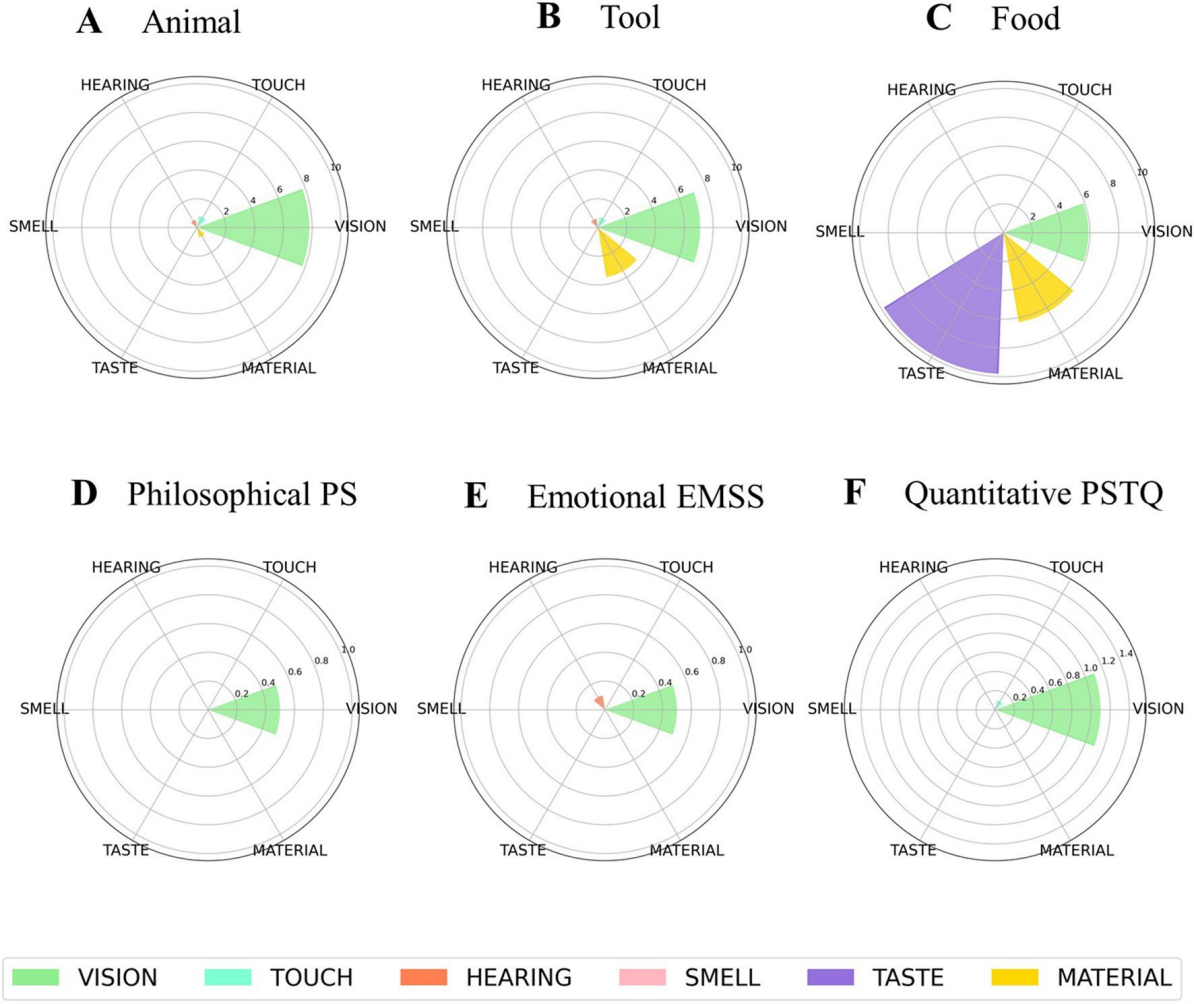
Polar plots for sentences including animals (**A**), tools (**B**), food (**C**), PS, philosophical-spiritual (**D**), EMSS, emotional-social (**E**), and PSTQ, physical-spatio-temporal-quantitative (**F**) concepts, showing the percentage of sensorimotor grounding dimensions, i.e., hearing (coral), touch (aquamarine), vision (light green), material (gold), taste (medium purple), smell (light pink).

**Figure 3:**
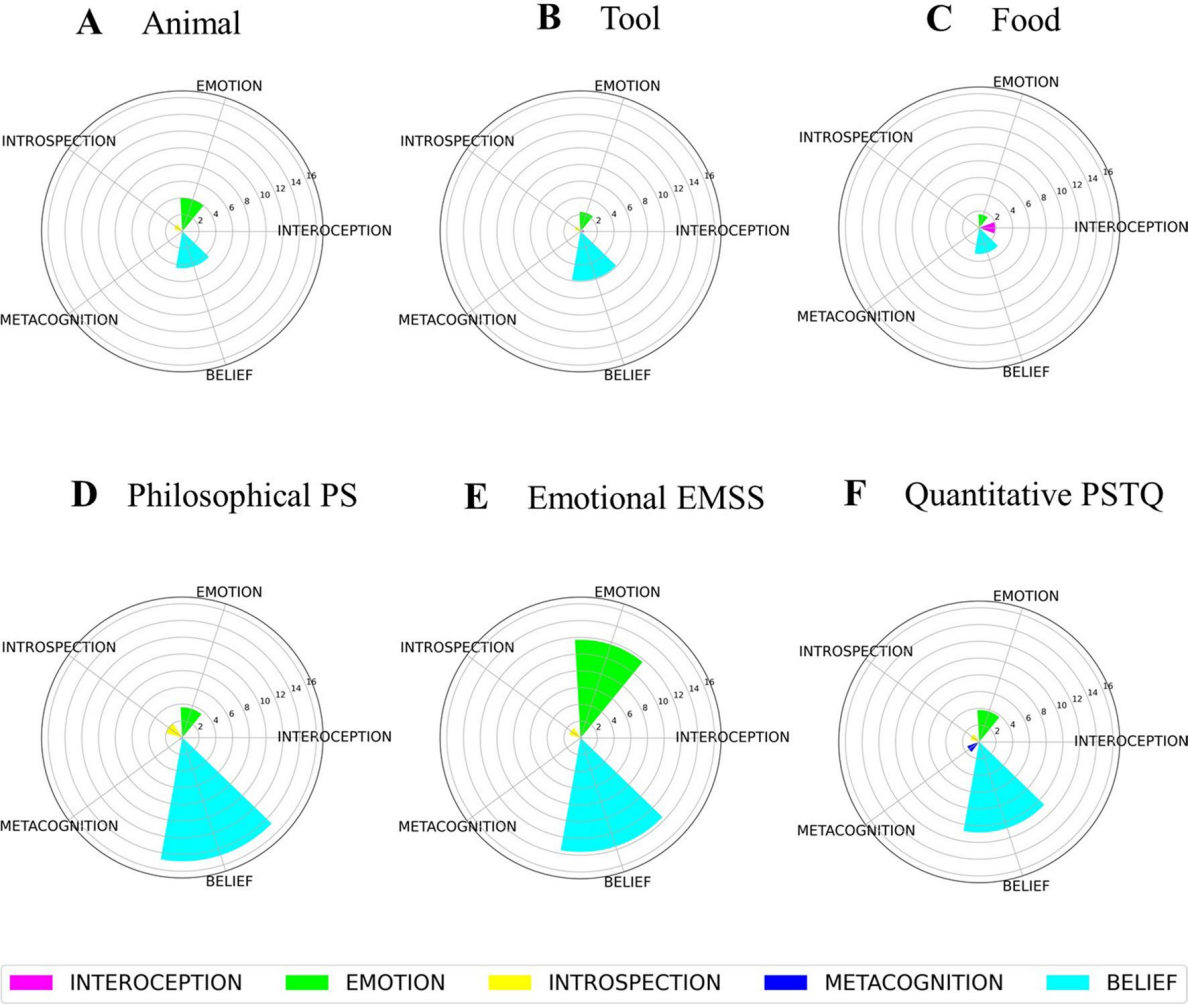
Polar plots for sentences including animals (**A**), tools (**B**), food (**C**), PS, philosophical-spiritual (**D**), EMSS, emotional-social (**E**), and PSTQ, physical-spatio-temporal-quantitative (**F**) concepts, showing the percentage of inner grounding dimensions, i.e., emotion (lime), interoception (fuchsia), belief (cyan), metacognition (blue), introspection (yellow).

Bayesian Generalized linear mixed models were applied to estimate the probability of different models' hypotheses, separately for each variable in ACs and CCs, and their subcategories (see “Analysis” below). The Bayes Factor (BF) for the Kind of concepts (i.e., emotional EMSS, philosophical PS, quantitative PSTQ, Animals, Food, Tool) and the Type of sentences (i.e., abstract, concrete) and Null Hypothesis models have been calculated for the model selection to each hypothesis (see Table 1). The subsequent analyses have been carried out according to the BF values. Table 2 shows a summary of the results. We report below the contrast analysis. This method allows us to compare the level of the factor on hypothesized differences between the ACs and CCs kinds. For each kind of concept (i.e., emotional EMSS, philosophical PS, quantitative PSTQ, Animals, Food, Tool) the frequency of coding variables is displayed in polar plots grouped according to our hypotheses on: conversation, turn-taking (Fig. 1), sensorimotor grounding (Fig. 2), inner grounding (Fig. 3). Polar plots showing the frequency of other variables are reported in Supplementary Materials.

**Table 1:** Bayes factor (BF) values for model comparison between the model carried out using the Type of sentences as the factor with the Null Hypothesis model (only intercept) (first column); the model carried out using the Kind of concepts as the factor with the Null Hypothesis (only intercept) (second column); the Type of sentences and the Kind of concepts models (last column).

Variables	BF (type of sentences) vs BF (null hypothesis)	BF (kind of concepts) vs BF (null hypothesis)	BF (kind of concepts) vs BF (type of sentences)
Abstract actions	1.25E+43	**6.44E+46**	5.11E+03
Concrete actions	6.45E+136	**2.77E+147**	4.27E+10
Associations	2.44E+00	**2.46E+03**	1.02E+03
Beliefs	2.04E+27	**1.51E+29**	7.42E+01
Emotions	7.28E+8	**1.39E+22**	1.92E+13
Events	2.56E+10	**6.08E+12**	2.48E+02
General statements	1.22E+24	**9.30E+25**	7.47E+01
Hearing^a^	**3.12E+03**	–	–
How-Questions	0.33	3.64E+28	**1.10E+29**
Interoception	1.56E+03	**2.49E+10**	1.58E+07
Introspection	2.91E+02	**3.04E+03**	1.02E+01
Material^a^	**2.99E+31**	–	–
Metacognition	6.92E+04	**8.80E+12**	1.25E+8
Number of questions	0.1	1.07E+07	**1.10E+08**
Non-perceptual evaluations	4.31	**9.68E+12**	2.25E+12
Number of target word repetition	1.51	**3.12E+14**	2.17E+14
Number of evoked contexts	**0.98**	0.07	0.07
Point of view	**0.28**	–	–
1st or 2nd point of view	–	**6.37**	–
1st & 2nd point of view	–	**7.60E+01**	–
2nd point of view	**0.34**	–	–
1st point of view	–	**8.23E+11**	–
3rd point of view	1.48	**1.36E+03**	9.29E+02
Smell^a^	**9.89E+01**	–	–
Space	3.12E+27	**5.93E+44**	1.88E+17
Subordinates	6.51	**6.88E+11**	1.08E+11
Taste	**9.98E+74**	–	–
Time	1.26E+04	**1.59E+10**	1.26E+06
Touch	7.19E+04	**5.00E+10**	6.71E+05
Turn-taking	0.24	5.54E+19	**2.36E+20**
Uncertainty expressions	5.87E+02	**1.33E+12**	2.20E+09
Vision	1.89E+33	**1.19E+34**	6.33
What-Questions	9.94E+14	**3.05E+51**	3.02E+36
When-Questions	1.57E+03	**8.02E+06**	5.23E+03
Where-Questions	3.25E+57	**2.54E+97**	7.92E+39
Who-Questions	6.65E+22	**1.73E+47**	2.62E+24
Why-Questions	0.35	1.67E+32	**4.80E+32**

The BFs values highlighting meaningful differences are in bold. The BF values in bold are those that show a highest difference between the models. This value has helped us in the choice of the models to consider for the analyses.

^a^Due to few elements in the variable only the comparison between Type of sentences and Null Hypothesis was carried out.

**Table 2:** Results of Bayesian linear mixed effect models.

Variables	Type of sentences		Kinds of concepts								
	Abstract	Concrete	EMSS	PS	PSTQ	Animal	Food	Tool	ACs vs. CCs	95% CI	PP
Uncertainty expressions			0.24	1.81	0.76	0.69	0	0.3	0.6	[0.2, 1.1]	**ACs > CCs, 99**
Number of questions			0.38	0.55	0.50	0.57	0.45	0.41	0.0	[− 0.04, 0.04]	ACs > CCs, 50
Number of target word repetition			2.5	5.1	2.5	6.2	5.4	1	− 0.8	[− 1.9, 0.1]	ACs > CCs, 3.6
Turn-taking			39.1	58.2	51.8	58.4	44.8	41.5	1.5	[− 1.4, 4.3]	**ACs > CCs, 83.6**
1st or 2nd point of view			40.1	35.3	32.8						
1st & 2nd point of view			10. 2	5.8	7.5						
1st point of view						9	18.7	7.4			
3rd point of view			0.72	0.13	0.48	0.6	0.25	0.13	0.11	[− 0.2, 0.5]	**ACs > CCs, 75.6**
General statements			8.5	10.6	6.9	3.3	1.7	2.3	6.2	[4.7, 7.9]	**ACs > CCs, 100**
Number of evoked contexts			5.5	5	5	4	4.5	4.4	0.87	[− 0.22, 1.95]	**ACs > CCs, 93**
Vision			0.5	0.5	1.1	7.8	5.9	7. 1	6.2	[5, 7.4]	**CCs > ACs, 100**
Touch			0	0	0.1	0.8	0.1	0. 72	0.5	[0.2, 1.0]	**CCs > ACs, 100**
Hearing			0.1	0	0	0.5	0	0.6	0.3	[0.08, 0.7]	**CCs > ACs, 99.9**
Taste	0	9.9							9.9	[8.7, 11.2]	**CCs > ACs, 100**
Smell	0	0.12							0.1	[4.5e−5, 3.04e−3]	**CCs > ACs, 99.6**
Materials			0	0	0	0.7	6.3	3.5	3.47	[2.5, 4.5]	**CCs > ACs, 100**
Space			1	0.2	0.6	7.6	0.9	7	4.6	[3.4, 5.9]	**CCs > ACs, 100**
Time			2.1	3.4	4	2.9	8.9	5.9	− 2.6	[− 3.8, − 1.5]	ACs > CCs, 0
Events			1.0	0.3	0.6	2.6	1.8	3.5	− 2	[− 2.9, − 1.2]	ACs > CCs, 0
Concrete actions			2.3	3.3	8.2	27.8	27.6	36.6	26	[23.4, 28.9]	**CCs > ACs, 100**
Abstract actions			6.3	11.1	6.6	0.5	1	1.2	7.14	[5.6, 8.8]	**ACs > CCs, 100**
Interoception			0.2	0	0.1	0.1	1.9	0.4	− 0.6	[− 1, − 0.3]	ACs > CCs, 0
Emotions			11. 7	3.6	3.8	4	1.5	2.3	3.8	[2.6, 5]	**ACs > CCs, 100**
Metacognition	0.63	0.04							0.6	[0.2, 1.1]	**ACs > CCs, 100**
Beliefs			13.6	14.8	10.8	4.4	3.1	5.9	8.6	[6.9, 10.6]	**ACs > CCs, 100**
Introspection			1.4	2	1.1	1	0.3	07	0.8	[0.3, 1.4]	**ACs > CCs, 99.9**
Associations			1.4	1.4	2.2	0.7	2.1	0.8	0.5	[0, 1.1]	**ACs > CCs, 95.8**
Subordinates	1.7	2.6							− 0.8	[− 1.6, 0.1]	**ACs > CCs, 100**
No Perceptual evaluations	9.1	7.3							1.8	[0.3, 3.4]	**ACs > CCs, 99**
Why-Questions			4.9	1.9	9.7	3.5	1	11	0.2	[− 0.9, 1.4]	**ACs > CCs, 68**
Who-Questions			12.2	1.32	0.88	0.99	0.55	0.88	4.7	[3.5, 5.8]	**ACs > CCs, 100**
What-Questions	14.13	6.89							7.2	[5.4, 9]	**ACs > CCs, 100**
Where-Questions			0.8	0.2	0	20.4	3	4.6	8.9	[7.6, 10.2]	**CCs > ACs, 100**
When-Questions			0.4	0.2	0	1	1.4	0.5	0.7	[0.3, 1.2]	**CCs > ACs, 100**
How-Questions			8.3	11.2	7.5	5	17.7	2.8	− 0.5	[− 2, 1]	CCs > ACS, 25.9

We showed the main probability of conditional effects according to the type of sentence (abstract, concrete) or kind of concepts (i.e., emotional EMSS, philosophical PS, quantitative PSTQ, Animals, Food, Tool) in each variable. We report the percentage of all variables for each kind of sentence, with the exception of the variables “number of questions” and “numbers of evoked contexts” where the count frequency is reported. This discrepancy is due to the fact that for the variables “number of questions” and “number of evoked contexts” we did not have a total numerical reference value, while for the others we had it, consequently we reported the percentages*.* For each variable, we report the comparative analysis between Abstract and Concrete sentences (ACs vs. CCs) and the 95% Credible Intervals (CI) and the probability of posterior observations major to zero (PP > 0) reported in percentage terms according to our hypothesis. The PPs values highlighting meaningful differences are in bold.

## Hypothesis 1. Conversation: uncertainty, number of questions, and target word repetitions

ACs elicited more *Uncertainty Expressions* than CCs. In particular, we found moderate evidence that PS sentences elicited more Uncertainty expressions than other ACs (1.4%; 95% CI [0.6, 2.5]. However, by inspecting the mean number of posterior observations major to zero (PP, Posterior Probability) we are 100% confident that the Uncertainty Expressions in PS sentences are more frequent compared to all other sentences. We found inconclusive evidence in support of the hypothesis that ACs evoke a higher *Number of Questions* than CCs. However, contrast analysis showed strong evidence that PS sentences elicited more questions than EMSS and PSTQ sentences (0.11; 95% CI [0.05, 0.17]; PP = 99.9%). We found inconclusive evidence for the hypothesis that *Target Word Repetitions* were more frequent with ACs than CCs. However, contrast analysis showed strong evidence that Target Word Repetitions were more frequent with the most abstract PS sentences compared to the other ACs (i.e., EMSS, PSTQ) (2.5%; 95% CI [1.1, 4.3]; PP = 99.9%).

## Hypothesis 2. Conversation: turn-taking

We found moderate/strong evidence that ACs generate more *Turn-Taking* (i.e., further interactions, turn of words) than CCs. Within ACs, we found strong evidence that turn-taking was more frequent with the most abstract PS sentences than EMSS and PSTQ sentences (12.7%; 95% CI [8.4, 17.1]; PP = 100%).

## Hypothesis 3. Conversation: Point of views and general statements

We found strong evidence for the hypothesis that ACs elicit more *General Statements* than CCs. In addition, contrast analysis showed strong evidence that General Statements were more frequent with PS sentences compared to PSTQ and EMSS sentences (2.9%; 95% CI [0.7, 5.5]; PP = 99.5%). Concerning the Points of view of participants, we found strong evidence that *1st or 2nd person Points of View* are more frequent with EMSS and PS sentences than PSTQ sentences (4.9%; 95% CI [0.9, 8.9]; PP = 99.1%), and moderate evidence that *1st and 2nd person Points of Views* were more frequent with EMSS and PS sentences than PSTQ sentences (0.6%; 95% CI [− 1.6, 2.6]; PP = 69.3%). We found strong evidence that 1st Point of View was more frequent with food and animal sentences than tool sentences (6.5%; 95% CI [4.0, 9.1]; PP = 100%) and moderate evidence that the *3rd Point of View* was more frequent with ACs than CCs. According to the Bayes Factor (BF, see Table 1), it was not possible to demonstrate the effect of sentences on overall Point of View and on 2nd Point of View.

## Hypothesis 4. Number of evoked contexts

We found strong evidence that the *Number of Evoked Contexts* was higher with ACs than CCs. In addition, contrast analysis showed inconclusive evidence for the hypothesis that PS and PSTQ sentences evoked a higher number of contexts than EMSS sentences (− 0.48, 95% CI [− 2.29, 1.18]; PP = 29%); and actually, it was more plausible that the effect was in the opposite direction as compared to the predicted one.

## Hypothesis 5. Produced features

### Sensorimotor grounding

We found strong evidence that CCs elicited more *Sensory* (i.e., vision, touch, hearing, taste, smell) and *Material features* than ACs (see Fig. 2).

Within CCs, contrast analysis showed inconclusive evidence that *Visual features* were more frequent with animal and food sentences than tool sentences (− 0.02; 95% CI [− 2.4, 1.8]; PP = 41.7%); moderate evidence that *Tactile features* were more frequent with tool sentences than other CCs (i.e., animal, food) (0.2%; 95% CI [− 0.2, 1]; PP = 85%), inconclusive evidence that *Tactile features* were more frequent with tool and food sentences compared to animals sentences (− 0.3%; 95% CI [− 1.2, 0.1]; PP = 6.2%); strong evidence that *Auditory features* were more frequent with animal and tool sentences than food sentences (0.5%; 95% CI [0.1, 1.2]; PP = 100%), and strong evidence for the hypothesis that, within CCs, *Material features* were more frequent with food and tools sentences compared to food sentences (4.2%; 95% CI [2.8, 5.7]; PP = 100%).

Within ACs, contrast analysis showed strong evidence for the hypothesis that the more concrete PSTQ sentences elicited more *Visual features* than other ACs (i.e., EMSS, PS) (0.6%; 95% CI [− 0.1, 1.4]; PP = 95.4%).

### Thematic relations: space, time, events, and actions

*Spatial features* were more frequent with CCs than ACs. In addition, contrast analysis showed strong evidence for the hypothesis that Spatial features were more frequent with tool sentences than other CCs (4.9%; 95% CI [3.1, 7]; PP = 100%). Within ACs, we found inconclusive evidence that Spatial features were more frequent with the most concrete PSTQ sentences compared to the other ACs (i.e., EMSS, PS) (− 0.02%; 95% CI [− 0.5, 0.6]; PP = 47.3%).

We found inconclusive evidence for the hypothesis that ACs elicit more *Temporal features* than CCs. However, contrast analysis showed strong evidence for the hypothesis that, within ACs, the most concrete PSTQ sentences evoke more Temporal features than other ACs (1.3%; 95% CI [− 0.2, 2.9]; PP = 95.9%). Within CCs, we found inconclusive evidence for the hypothesis that animal sentences evoke more Temporal features compared to other CCs (− 4.3%; 95% CI [− 6.1, − 2.6]; PP = 0%), and actually, it was more plausible that the effect was in the opposite direction as compared to the predicted one. We found inconclusive evidence in support of the hypothesis that *Events* were more frequent with ACs than CCs.

*Concrete actions* were more frequent with CCs than ACs. Within CCs, contrast analysis showed strong evidence that Concrete Actions were more frequent with food and tools sentences than animal sentences (4.3%; 95% CI [0.3, 8.1]; PP = 98.3%). Within ACs, contrast analysis showed strong evidence for the hypothesis that PSTQ sentences elicit more Concrete Actions than other ACs (i.e., EMSS, PS) (5.3%; 95% CI [3.4, 7.6]; PP = 100%).

*Abstract Actions* were more frequent with ACs than CCs. In addition, contrast analysis showed inconclusive evidence in support of the hypothesis that Abstract Actions were more frequent with PSTQ sentences than PS and EMSS sentences (− 2.1%; 95% CI [− 4.21, 0]; PP = 2.4%), and actually, it was more plausible that the effect was in the opposite direction. Consistently, we found strong evidence that Abstract Actions were more frequent with PS and EMSS sentences than PSTQ sentences (2.1%; 95% CI [0, 4.21]; PP = 97.6%).

### Inner grounding: interoception, emotions, metacognition, beliefs, and introspections

We found inconclusive evidence in support of the hypothesis that *Interoceptive features* were more frequent with ACs than CCs. However, contrast analysis showed moderate/strong evidence that, within ACs, Interoceptive features were more frequent with EMSS sentences than PS and PSTQ sentences (0.14%; 95% CI [0, 0.6]; PP = 88.3%). Within CCs, we found strong evidence that Interoceptive features were more frequent with food sentences compared to tool and animal sentences (1.6%; 95% CI [0.7, 2.7]; PP = 100%).

Overall, we found strong evidence that ACs evoke more *Emotions, Metacognition features, Beliefs,* and *Introspection* than CCs (see Fig. 3).

Within ACs, contrast analysis showed strong evidence for the hypothesis that *Emotions* were more frequent with EMSS sentences than other kinds of ACs (i.e., PS, PSTQ) (8%; 95% CI [5.6, 10.6]; PP = 100%)*;* inconclusive evidence for the hypothesis that the most abstract PS sentences evoke more *Metacognitive features* than other ACs (i.e., EMSS, PSTQ) (− 0.05%; 95% CI [− 1.13, 0]; PP = 2.3%); strong evidence for the hypotheses that the most abstract PS sentences evoke more *Beliefs* than the EMSS and PSTQ sentences (2.6%; 95% CI [0.2, 5.6]; PP = 96.3%), and that Beliefs were more frequent with PS and EMSS sentences compared to the less abstract PSTQ sentences (3.4%; 95% CI [0.7, 6.2]; PP = 99.3%). Finally, we found strong evidence that PS sentences elicit more *Introspective* states than EMSS and PSTQ sentences (0.7%; 95% CI [− 0.1, 1.8]; PP = 95.2%), and that Introspection was more frequent with PS and EMSS sentences than the less abstract PSTQ sentences (0.6%; 95% CI [− 0.2, 1.5]; PP = 94.1%).

### Other: associations, subordinates, and non-perceptual evaluations

We found strong evidence that *Associations* were more frequent with ACs than CCs. However, contrast analysis showed inconclusive evidence for the hypothesis that, within ACs, Associations were more frequent with PS sentences compared to PSTQ and EMSS sentences (− 0.4%; 95% CI [− 1.3, 0.4]; PP = 15.6%). We found inconclusive evidence in support of the hypothesis that *Subordinates* were more frequent with ACs than CCs (− 0.8%; 95% CI [− 1.6, 0.1]; PP = 1%), and strong evidence that *Non-perceptual evaluations* were more frequent with ACs than CCs.

## Hypothesis 6. Conversation: kinds of questions

*Why questions* were slightly more frequent with ACs than CCs. In addition, contrast analysis showed inconclusive evidence for the hypothesis that, within ACs, PS and EMSS sentences evoke more Why Questions than PSTQ sentences (− 6.2; 95% CI [− 8.7, − 4.1]; PP = 0%), and actually it was more plausible that the effect was in the opposite direction as compared to the predicted one. Consistently, we found strong evidence that Why Questions were more frequent with PSTQ sentences compared to PS and EMSS sentences (6.2%; 95% CI [4.1, 8.7]; PP = 100%).

*Who questions* were more frequent with ACs than CCs. In addition, contrast analysis showed strong evidence for the hypothesis that, within ACs, EMSS sentences evoke more Who Questions than PS and PSTQ sentences (10%; 95% CI [7.5, 12.9]; PP = 100%).

*What questions* were more frequent with ACs than CCs. Instead, W*here* and *When questions* were more frequent with CCs than ACs. Within CCs, contrast analysis showed strong evidence that Where Questions were more frequent with animal and tools sentences than food sentences (9.4%; 95% CI [7.4, 11.5]; PP = 100%).

*How questions* were not more frequent with CCs than ACs as we assumed. However, contrast analysis showed strong evidence that, within CCs, How Questions were more frequent with food and tool sentences than animal sentences (5.2%; 95% CI [3.1, 7.3]; PP = 100%). Within ACs, contrast analysis showed inconclusive evidence for the hypothesis that How Questions were more frequent with EMSS sentences compared to PS and PSTQ sentences (− 1%; 95% CI [− 3.3, 1.3]; PP = 19%); and that PSTQ sentences evoke more How Questions than EMSS and PS sentences (− 2.2%; 95% CI [− 4.5, 0]; PP = 2.7%). Consistently, we found strong evidence that PS sentences evoke more How Questions compared to the other kinds of ACs (3.3%; 95% CI [0.9, 5.9]; PP = 99.6%).

### Exploratory analyses on empathy

We ran correlation analyses to explore the relationship between the results of the conversational task and the dispositional empathy of participants, detected using the IRI scale^42^. Among the four subscales included in the IRI survey, we focused on the perspective-taking (PT) and empathic concern (EC) subscales that tap separate facets of empathy particularly relevant for our purposes. The PT subscale measures the reported tendency to adopt the psychological point of view of others in everyday life (e.g., "I sometimes try to understand my friends better by imagining how things look from their perspective"). In contrast, the EC subscale assesses the tendency to experience feelings of sympathy and compassion for unfortunate others (e.g., "I often have tender, concerned feelings for people less fortunate than me").

Specifically, we tested whether the main PT and EC scores of each participant correlated with the following variables: Turn-Taking, Agreement, Emotions, Points of View (1st, 2nd, 3rd, and 1st and 2nd person), Why and How questions. Finally, we tested whether the number of words produced by participants varied across different sentences. We investigated whether such correlations are different in CCs and ACs, and their sub-categories.

### Exploratory analyses results

Because of the frequency nature of our dependent variables, we conducted our analysis using Spearman’s Rho correlation. Table 3 shows a summary of the results for each variable.

**Table 3:** Spearman rank correlation between the empathic concern (EC) and perspective-taking (PT) subscale for the selected variables across abstract and concrete sentences and sub-type of concrete sentences (i.e., animals, foods, tools) and abstract sentences (i.e., emotional EMSS; philosophical PS; quantitative PSTQ).

Variable	Abstract		Concrete		Animals		Foods		Tools		Emotional EMSS		Philosophical PS		Quantitative PSTQ	
	EC	PT	EC	PT	EC	PT	EC	PT	EC	PT	EC	PT	EC	PT	EC	PT
**Turn-taking**																
*Rho*	− 0.021	− 0.028	0.031	0.052	0.040	0.078	0.034	0.043	0.021	0.037	− 0.013	− 0.041	− 0.039	− 0.001	− 0.013	− 0.042
*p*	0.305	0.175	0.127	**0.010**	0.254	**0.028**	0.333	0.227	0.553	0.295	0.716	0.251	0.269	0.969	0.719	0.232
**Agreement**																
*Rho*	− 0.002	− 0.011	− 0.040	− 0.051	− 0.0133	− 0.062	− 0.065	− 0.089	− 0.043	− 0.012	− 0.018	0.013	− 0.001	− 0.029	0.014	− 0.020
*p*	0.915	0.592	0.052	0.**012**	0.710	0.078	0.067	**0.012**	0.224	0.725	0.606	0.704	0.969	0.407	0.686	0.567
**Emotions**																
*Rho*	0.059	0.025	0.015	0.016	0.043	0.078	− 0.045	− 0.005	0.029	0.028	0.090	0.027	0.038	0.024	0.036	0.026
*p*	**0.004**	0.218	0.466	0.438	0.224	0.558	0.203	0.878	0.418	0.423	**0.011**	0.453	0.283	0.502	0.306	0.460
**Why-Questions**																
*Rho*	− 0.065	0.002	− 0.027	− 0.044	0.019	− 0.078	− 0.093	− 0.066	− 0.038	− 0.025	− 0.053	− 0.001	− 0.022	0.007	− 0.101	0.001
*p*	**0.001**	0.904	0.189	**0.031**	0.583	**0.027**	**0.008**	0.063	0.280	0.489	0.136	0.980	0.534	0.837	**0.004**	0.969
**How-Questions**																
*Rho*	0.050	− 0.016	0.018	0.033	0.021	0.078	0.017	0.034	0.020	− 0.013	0.119	− 0.011	0.023	− 42	0.007	0.009
*p*	**0.015**	0.445	0.384	0.105	0.549	**0.028**	0.627	0.344	0.575	0.721	**0.001**	0.767	0.507	0.234	0.840	0.793
**Point of view**																
*Rho*	0.044	0.053	0.050	0.035	0.022	− 0.010	0.062	0.032	0.066	0.081	0.061	0.079	0.012	0.021	0.059	0.055
*p*	**0.032**	**0.010**	**0.014**	0.091	0.530	**0.777**	0.082	0.368	0.064	**0.022**	0.085	**0.026**	0.727	0.552	0.098	0.119
**1st Point of view**																
*Rho*	0.035	0.019	0.041	− 0.077	0.019	− 0.109	0.030	− 0.040	0.084	− 0.100	0.045	0.049	0.050	0.028	0.008	− 0.022
*p*	0.087	0.352	**0.043**	**0.001**	0.585	**0.002**	0.399	0.265	**0.017**	**0.005**	0.208	0.164	0.161	0.423	0.812	0.530
**2nd Point of view**																
*Rho*	− 0.005	0.022	0.023	0.039	0.033	0.056	0.020	0.000	0.018	0.061	− 0.004	0.022	− 52	0.019	0.043	0.024
*p*	0.794	0.287	0.259	0.054	0.347	0.117	0.568	0.999	0.614	0.008	0.910	0.533	0.143	0.595	0.222	0.503
**3rd Point of view**																
*r*	− 0.008	0.015	− 0.018	− 0.028	− 0.008	− 0.039	− 0.019	− 0.036	− 0.047	− 0.005	0.007	0.063	0.004	− 0.026	− 0.035	− 0.021
*p*	0.697	0.476	0.375	0.169	0.829	0.273	0.598	0.314	0.186	0.881	0.852	0.077	0.900	0.465	0.326	0.546
**1st and 2nd Point of view**																
*Rho*	0.044	0.030	0.026	0.061	− 0.016	− 0.014	0.046	0.089	0.037	0.088	0.061	0.039	0.043	− 0.008	0.028	0.054
*p*	**0.031**	0.143	0.202	**0.003**	0.645	0.701	0.195	**0.012**	0.302	**0.013**	0.086	0.274	0.230	0.828	0.423	0.129

In bold are reported significant correlation (p < .05).

EC sub-scale, more emotionally connoted, correlated mostly with ACs and their kinds. In particular, the positive correlation between EC subscale and EMSS sentences for emotions, how-questions, and 1st and 2nd points of view suggests that more empathic individuals may be more sensitive to emotions and keener to adopt others’ perspectives asking questions about how they feel. Within CCs, the EC sub-scale correlated with tools in first-person pronouns; hence, such a personal involvement concerns tools, likely because of their link with action.

PT sub-scale mainly correlated with CCs and their subkinds. The positive correlation of the PT scale with the further turn-taking for concrete sentences concerns mainly animals. Unsurprisingly, the level of agreement correlated negatively with the PT subscale for food sentences, for which participants frequently express their own tastes. In addition, the negative correlation between PT subscale and why-questions for animals and foods suggest that people who adopt the psychological point of view of others are less prone to inquire about common actions involving everyday objects/entities. Consistently, we found a negative correlation between the PT subscale in 1st person and a positive correlation in 2nd person and 1st and 2nd person combined, especially with tools and foods, possibly due to their link with interactive and joint actions. However, these findings do not allow drawing strong conclusions because the Spearman’s correlation indicates only weak relationship.

Finally, we used the Wilcoxon Signed-Ranks test to explore the Number of Words produced by participants. We found no differences between the produced texts to ACs and CCs (*Z* = 1.666, *p* = 0.067). However, according to Friedman's test the sub-categories of ACS and CCs differ significantly in the produced numbers of words (χ^2^_F_ (5) = 20.03, *p* < 0.001). Participants tend to produce longer responses to sentences including food items (*Mdn* = 3.84) and EMSS concepts (*Mdn* = 3.86) than animals (*Mdn* = 2.81) (*Z* = − 1.056, *p* = 0.007; *Z* = − 1.038, *p* = 005, respectively). No other pairwise comparison reached the significance.

## Discussion

The results are in line with the predictions. CCs and ACs differ along various dimensions. CCs evoke more sensorimotor properties, ACs elicit more inner properties (emotions, beliefs, etc.). Crucially, CCs and ACs also differ in the conversational dynamics they elicit. Furthermore, exploratory analyses confirmed the differences between the various concept kinds. We will first summarize the main differences between CCs and ACs, and then between concept kinds.

### ACs

Compared to CCs, ACs generate more uncertainty expressions, a higher number of cases of 1st and 2nd person point of views, and evoke a higher number of contexts. Furthermore, they yield more associations, more inner processes (emotions, beliefs, introspections), more general statements. In addition, they evoke more why and who questions. Finally, the correlation between the Empathic Concern subscale and ACs, especially EMMS, for emotions and how-questions suggests a higher personal engagement with ACs, confirmed by the correlations with 1st and 2nd point of view. Some of these dimensions have been identified in previous norming, ratings, and feature production studies: for example, Villani et al.^17,28^ and Barsalou et al.^25^ stressed the role of inner grounding; Kousta et al.^32^ the importance of emotions; Barsalou and Wiemer-Hastings^30^ and Barca et al.^43^, the role of free associations, and introspections; Schwanenflugel et al.^9^, the association with various contexts. However, some dimensions are completely new since they characterize ACs in a simulated direct interaction. As predicted, ACs generate more uncertainty, as testified by the correspondent expressions, and, possibly as a consequence of this, lead to more interactive exchanges, assessed through the higher presence of 1st and 2nd person point of views and the higher numbers of turns. They elicit more generalizations and questions linked to possible underlying mechanisms (why) and agents (who).

### CCs

Compared to ACs, CCs yield more sensory properties, materials, spatial expressions, and concrete actions. These dimensions confirm that CCs are firmly grounded in the sensorimotor system; unlike previous studies, we find these properties using a simulated interaction rather than isolated words. Crucially, we also find that CCs elicit more questions about the spatial and temporal context (what, where, and when). Finally, the finding that the CCs and the Perspective Taking subscale correlated positively for 1st and 2nd point of views and correlated negatively for why-questions and 1st person perspective is an index that CCs are rooted on common ground knowledge that requires fewer specifications.

### Kinds of ACs

While some dimensions, like metacognition, do not differ across the kinds of ACs, a major opposition exists between the more abstract PS and the more concrete PSTQ concepts. Consistently, compared to the two other kinds of ACs, PS concepts elicit more uncertainty in conversation (uncertainty expressions, questions, and repetitions) and a more interactive dialogue (more turns). Inner grounding is stronger (more beliefs and introspections), and the tendency to produce general statements is more marked. PS concepts evoke more points of view and abstract actions than PSTQ ones. In contrast, PSTQ concepts evoke more visual properties and concrete actions than other abstract kinds. Curiously, they also elicit more temporal features and why-questions, but this is likely a matter of the content they convey, being often scientific concepts. EMSS concepts are in the middle in terms of abstractness. Consistently, they evoke fewer points of view than PS, and more abstract actions than PSTQ concepts. Crucially, they are characterized for interoception (e.g.,^6,17^), and they yield more who-questions, testifying the interest for the person who experiences emotions.

### Kinds of CCs

Our results allow us to frame the kinds of CCs in a novel way. The personal involvement, as testified by 1st person statement, is stronger with animals and food, while where-questions interest more animals and tools, likely because of the possible variety of their contexts. Notably, food evokes both perceptual (materials) and bodily experiences (interoception); animals and tools elicit auditive properties.

## Conclusions

This study allows drawing three main conclusions.

The first is that the abstract/concrete distinction is an important one. CCs and ACs cannot be characterized as extremes of a continuum but as a collection of different points in a multidimensional space. Some of these dimensions have been intensively investigated. For example, classical theories emphasized the role of imageability for CCs and the higher number of contexts for ACs; recent views underlined the importance of emotions and inner grounding for ACs. But, crucially, other dimensions we identified are entirely novel, deriving from concepts’ use in a simulated conversational exchange. ACs induce higher conversational uncertainty, elicit why and who questions, and more interactive exchanges (use of the second person); conversely, CCs yield more what, where, and when questions.

The second is that ACs and CCs are not holistic categories but incorporate differently characterized kinds. Within ACs, the most concrete PSTQ oppose to the most abstract PS concepts. We identified both the content of each kind and the specificities linked to the use of the corresponding word. EMSS concepts differ from other kinds, both in terms of their content—the strong role of interoception—and their role in the conversation.

It should be noted that our study did not aim to compare the distinction of abstract-concrete concepts as a continuum vs. discrete categories. However, we believe that by showing that different dimensions, both semantic and pragmatic, weigh differently depending on the kind of concepts/sentences, we demonstrate that concepts are not arranged along a continuum between the two extremes defined only by the concreteness/abstractness dimension. Moreover, our results further corroborate the idea that concepts are multidimensional and multifaceted constructs. When considered as broad categories, concrete concepts are primarily grounded in sensorimotor experiences, and abstract concepts are mostly grounded in inner and social-linguistic experiences. However, at the more fine-grained level, the role of some dimensions overlaps between different types of concepts. For example, interoceptive contents characterized both food and emotional concepts, sensorimotor proprieties (visual and actions-related) are associated with tools and abstract physical-quantitative concepts.

The third is that investigating concepts in everyday use allows detecting their richness and flexibility. A possible limit in generalizing our results is that they concern a simulated conversational exchange. We should design new methods to investigate concepts in real-time interactions, benefiting from insights from pragmatics and using social interactive tasks. The absence of studies adopting interactive methods in the investigation of abstract concepts is particularly striking in light of the spread of interest in the role of social interaction in a variety of fields. For example, research on basic cognitive processes has recently highlighted that some specific effects on spatial representation and attentional processing emerged only when participants are sharing a task (e.g.,^44–46^, for a review, see^47^). The last 20 years have seen a proliferation of studies on sensorimotor communication and signalling (review^48^) and on various forms of synchronization occurring during conversations (e.g.,^49^), intended as a form of joint action^50,51^. In neuroscience, the development of new, sophisticated techniques, like hyperscanning, has allowed the focus on interactive aspects during conversation (meta-analysis^52^). Curiously, this interest in interactive aspects has not included the investigation of conceptual representation (for exceptions see^38,39^). In a recent paper, Barsalou et al.^25^ argued that most studies in this field used single, decontextualized words and stated that we need to study concepts in situated action. We agree and think we need to go even further and start investigating concepts in situated interactions.

## Method

The hypotheses, experimental procedures, and data analysis have been specified in a pre-registration available at https://osf.io/6mkc7. Data has been collected after the pre-registration.

## Materials

Stimuli consisted of 60 sentences composed of a verb and a concept noun. Concept nouns included 30 abstract concepts and 30 concrete concepts used in a previous study^17^. The set of stimuli consisted of 3 sub-categories of concrete concepts, i.e., ten tools (e.g., “hammer”, “umbrella”, “fork”), ten animals (e.g., “lion”, “dog”, “cow”), and ten food items (e.g., “banana”, “tomato”, “carrot”), and three sub-categories of abstract concepts, i.e., ten philosophical-spiritual (PS, e.g., “moral”, “destiny”, “salvation”), ten physical-spatio-temporal-quantitative (PSTQ, e.g., “area”, “number”, “acceleration”), and ten emotional-social concepts (EMMS, e.g., “shame”, “joy”, “conflict”). We controlled the frequency of use of target nouns. Specifically, the subgroups of abstract and concrete words were balanced for classical psycholinguistic variables, including the absolute frequency (concrete food, tools, animals: *F*(2,27) = 0.536; *MSE* = 3758.196; *p* = 0.591; ƞp2 = 0.038; abstract PS, EMSS, PSTQ: *F*(2,27) = 1.855; *MSE* = 72,376.078; *p* = 0.18; ƞp2 = 0.121) and relatively frequency (concrete food, tools, animals *F*(2,27) = 0.694; *MSE* = 178.537; *p* = 0.508; ƞp2 = 0.049; abstract PS, EMSS, PSTQ: *F*(2,27) = 1.817; *MSE* = 4541.619; *p* = 0.18; ƞp2 = 0.119) based on CoLFIS, a lexical database of written Italian^53^ (further details of psycholinguistic variables of target nouns are available at https://osf.io/rx85h/, and Villani et al*.*^28^ database).

For each of the selected concepts, we created a sentence in the Italian language. Each sentence was constructed by pairing a verb in present perfect tense with the concept noun (e.g., Ho fatto una torta/I made a cake; Ho pensato al destino/I thought about destiny). All sentences were declarative statements in the first person, balanced for definitive and indefinite articles and length (from min. 20 to max. 26 letters). See Supplementary Materials for the full list of sentences.

### Participants

The choice of our sample size was guided by reference to a previous study in literature in which similar measurements and statistical analyses are used (N = 62^38^). We recruited 92 native Italian speakers through Qualtrics survey software among students of the Cognitive Psychology course and researchers of the University of Bologna, who were asked to disseminate the survey to colleagues or acquaintances. Participants with incomplete data were excluded (n = 12). The final sample consisted of 80 participants (59 female, Mage = 26.3, SDage = 5.9). The study was approved by the Ethical Committee of the University of Bologna and fulfilled the ethical standard procedure recommended by the Italian Association of Psychology (AIP) and conformed to the Declaration of Helsinki. All participants were naïve as to the purpose of the experiment and gave their informed consent to participate in the study.

### Procedure

The study was implemented as an online questionnaire in Qualtrics and consisted of three parts: (1) conversational task, (2) debriefing ratings, and (3) the Interpersonal Reactivity Index (IRI^40^; Italian version, see^54^).

**Figure 4:**
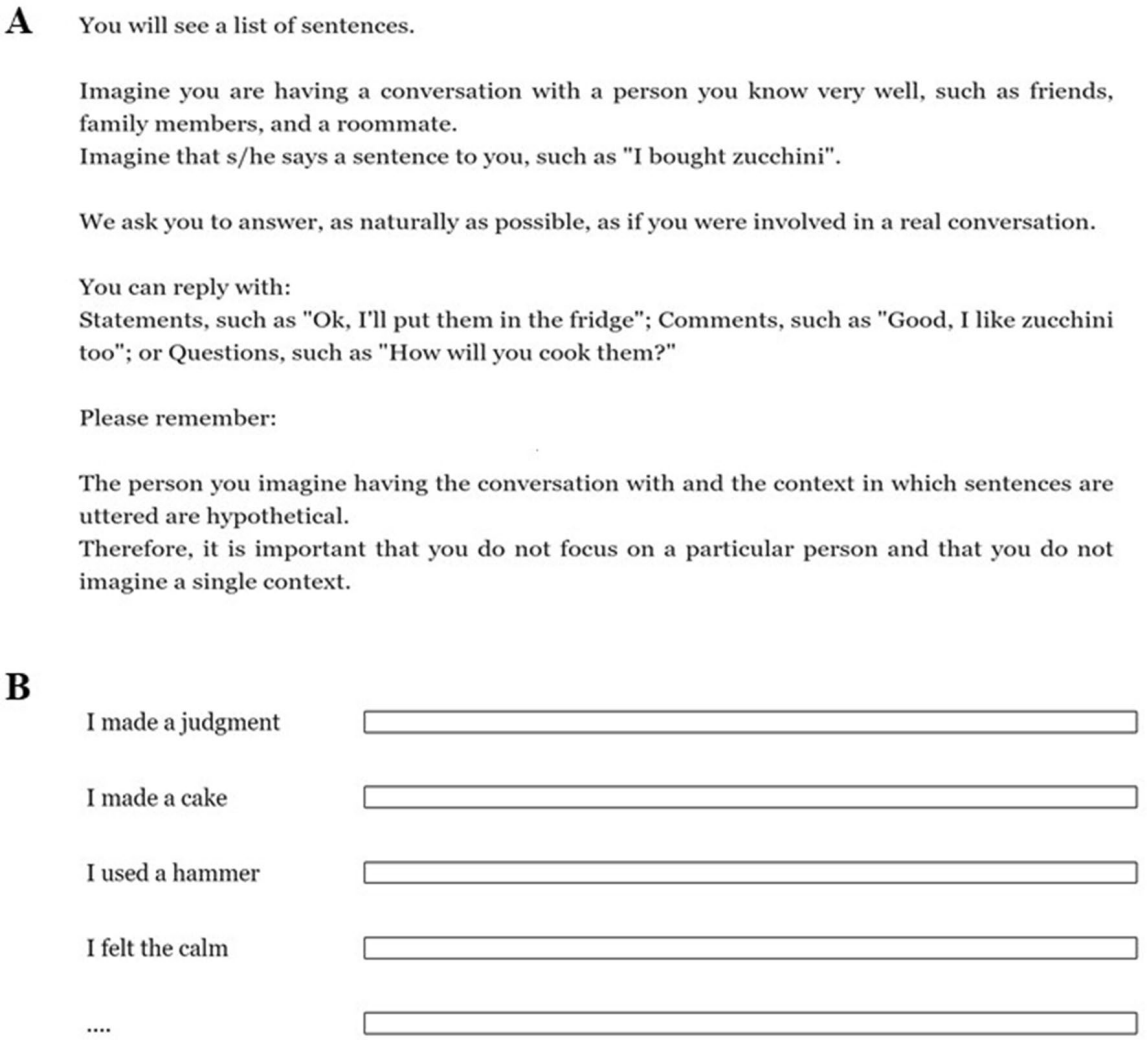
Instructions provided to the participants (**A**) and the Qualtrics interface with examples of four sentences used in the study (**B**).

In the conversational task, 60 written sentences were presented in random order. Participants were asked to respond through written language production, simulating a conversational exchange with another person. Specifically, participants saw a list of sentences; for each sentence they were asked to imagine a natural conversation with a familiar person who uttered the sentence and to write their own response as naturally as possible. Participants were invited to avoid focusing on a single person or situation during the task. No character limit was imposed. Figure 4 reports both the instructions provided to participants and the Qualtrics interface used in the experimental task.

In the debriefing ratings, participants were asked to rate their general comprehension of the task and how much they felt involved in a real conversation using 7-point scales ranging from 1 = “not at all” to 7 = “extremely”. Finally, they indicated which sentences they had had more doubts about answering.

In the last part of the questionnaire, participants completed the IRI survey, a 28-item self-report measure of empathy. It consists of four subscales with seven items measured on a 5-point scale ranging from 0 = “does not describe me well” to 4 = “describes me very well”. Each subscale measured different dimensions of dispositional empathy: the Perspective Taking (PT) assesses cognitive empathy, or the tendency to adopt the psychological point of view of others spontaneously; the Empathic Concern (EC) assesses emotional empathy, or the other-oriented feelings of sympathy and concern for unfortunate others; the Fantasy (FS) taps the respondent's tendency to transpose oneself into feelings and actions of fictional situations imaginatively; and the Personal Distress (PD) measures the tendency to experience anxiety and unease in response to other’s suffering^40^.

### Sentences coding

37 category codes (see Supplementary Materials) were used to best capture the type of features the participants produced with each sentence. Coding categories were adapted from Barsalou and Wiemer-Hastings^30^ and Zdrazilova et al*.*^38^. Two independent researchers, one of whom blind to the aims of the study, coded the produced text. Reliability among the coders was 96%. The cases of disagreement were solved through consensus after discussion together with a third judge.

### Debriefing responses

On average, participants declared to have correctly understood the task (M = 3.5; SD = 1) and to have simulated a real conversation (M = 3.7; SD = 0.8). Most of the sample showed moderate (34%) and good comprehension of the sentences used in the task (31%), 17% extreme, and only 16% poor comprehension. Half of the sample (51%) reported being very involved in natural speech, 27% moderately involved, 12% extremely involved, and only 9% felt slightly involved. Finally, participants reported more uncertainty when responding to sentences related to abstract concepts (40%) compared to concrete ones (20%). Within concrete concepts, they had major doubts about sentences related to animals (27.5%). Notice that the last percentages on uncertainty were calculated based on the responses to an open-ended question, in which participants were free to express doubts about one or more sentences used in the task.

### Analysis

Bayesian Generalized linear mixed models were applied to estimate the probability of different models' hypotheses. The Bayesian approach determines the probability that a model’s parameters take on different values, given the observed data. According to Bayes’ theorem, this is the combination of our prior expectations and the likelihood that we would have observed our data given different parameter values. Thus, functions describing the prior and the likelihood are combined to create a posterior density function. This is then sampled, and the resulting sample can be used to establish the 95% credible intervals: the range of values with a 95% probability of containing the true value for a given parameter. When a given parameter’s credible interval does not include zero, we consider it significantly different from zero and worth interpreting.

The Bayes factor (BF) is the ratio of the probabilities of the data in models 1 and 2 and indicates how much the prior odds change, given the data^55^. Conventionally, for converting the magnitude of the BF to a discrete decision about the models is that there is “substantial” evidence for model 1 when the BF exceeds 3.0 and, equivalently, “substantial” evidence for model 2 when the BF is less than 1/3^55^. In the present study, before proceeding with the inferential analyses, for each output variable, we selected the model with the highest Bayes Factor among those having substantial evidence. This procedure allows us to compare different models and different hypotheses and to choose the one for which we have the greatest evidence. Bayesian Generalized linear mixed models were applied separately on each outcome variable: uncertainty expressions, number of questions, repetitions of the target words, turn-taking, point of view (1st, 2nd, 3rd, 1st or 2nd, 1st and 2nd person perspective), number of evoked contexts, general statements, perceptual evaluations on vision, touch, hearing, smell, taste, materials/components, space, time, events, concrete actions, abstract actions, interoception, emotion, metacognition, belief/intentions, introspection, associations, subordinates, non-perceptual evaluations, why-questions, who-questions, where-questions, what-questions, when-questions, how-questions. A total of 37 analyses were developed.

In each model, the predictors were the Type of sentences (Abstract vs. Concrete) and the Kind of concepts. We had three kinds of ACs—Philosophical-Spiritual (PS), Physical-Spatio-Temporal-Quantitative (PSTQ), and Emotional-Mental State-Social (EMSS), three kinds of CCs—Tool, Food, Animals. We decided to use either the Kind of concepts or the Type of sentences as a predictor because these two factors are strongly correlated: it was therefore not possible to include both in the same model. In detail, when the Kind of concepts had zero or very few elements, the Type of sentences was preferred as a predictor. See Table 2. Further details of the model’s convergence and suitability of effective sample size are available as Supplementary Materials.

The Type of sentences and the Kind of concepts were the within-subject factors. Models included random subject intercepts. Random effects help generalize results beyond a particular set of subjects; accounting for subject-level variation (see^38^). Bayes factor and credible interval were used to make inferences. Regarding the variables “number of questions” and “number of evoked contexts” that had a count response outcome, models with Poisson distribution with logistic link function were developed, whereas for all other variables models with Binomial distribution with logit link function were carried out. Therefore, the results referring to “number of questions” and “number of evoked context” have been reported as count frequency values while all the other variables, being dichotomous, could be analyzed as percentage.

For models with Type of sentences and Kind of concepts factors, analyses were run computing four sampling chains, each with 10,000 iterations. For each chain, the first 4000 iterations are treated as warmups, resulting in 24,000 posterior samples. In addition, for a better sampler’s behavior, adapt_delta and max_treedepth parameters were set to 0.99 and 15, respectively. For the Null Hypothesis model or only intercept model, analyses were run computing four sampling chains, each with 5000 iterations. For each chain, the first 2000 iterations are treated as warmups resulting in 12,000 posterior samples. In addition, for a better sampler’s behavior, adapt_delta and max_treedepth parameters were set to 0.99 and 10, respectively.

Due to the lack of previous literature on the topic, models were fit using flat priors for fixed and random effects. All models were seen as reliable, reaching convergence with an R.hat that is the potential scale reduction factor on split chains equal to 1.00 and with suitable effective sample size measures evaluated with Bulk_ESS and Tail_ESS. Finally, we used a contrast method to explore the hypnotized differences between the ACs and CCs kinds. The analyses were carried out using R (version 4.0.3^56^); data processing was also carried out in part using ‘openxlsx’^57^, ‘dplyr’^58^, ‘lattice’^59^, ‘brms’^60^; this package allows fitting Bayesian mixed-effects models using the Stan programming language; ‘bayesplot’^61^; ‘gridExtra’^62^ and ‘repmod’^63^. The bar charts on polar axis graphs were carried out by using Python language (version 3.8) and *Matplotlib* and *Numpy* libraries.

## Supplementary Information

Supplementary Information 1.

Supplementary Information 2.

Supplementary Information 3.

Supplementary Information 4.

## Data Availability

All data and scripts are available at https://osf.io/mzaxw/.
