# Supplementary material for: Abstract and concrete concepts in conversation
Source: Sci Rep. 2022 Oct 20;12:17572. doi: 10.1038/s41598-022-20785-5 (PMC9584910; doi:10.1038/s41598-022-20785-5)
Supplement: Supplementary file 1 — Supplementary Information 1. [file 41598_2022_20785_MOESM1_ESM.docx]

**Tab1. List of concrete sentences**

| **Category** | **Italian Word** | **English Word** | **Concrete Sentence** | **Lenght** |
| --- | --- | --- | --- | --- |
| Foods | banana | banana | Ho mangiato una banana / *I eat a banana* | 23 |
|  | carota | carrot | Ho lessato le carote / *I boiled the carrots* | 20 |
|  | uva | grapes | Ho acquistato dell'uva / *I bought the grapes* | 22 |
|  | fragola | strawberry | Ho comprato le fragole / *I bought strawberries* | 22 |
|  | fungo | mushroom | Ho raccolto un fungo / *I picked up a mushroom* | 20 |
|  | melanzana | eggplant | Ho tagliato la melanzana / *I cut the eggplant* | 24 |
|  | peperone | pepper | Ho cucinato un peperone / *I cooked a pepper* | 23 |
|  | pomodoro | tomato | Ho condito il pomodoro / *I seasoned the tomato* | 22 |
|  | torta | cake | Ho preparato una torta / *I made a cake* | 22 |
|  | zucca | pumpkin | Ho decorato una zucca / *I decorated a pumpkin* | 22 |
|  |  |  |  |  |
| Tools | lampada | lamp | Ho acceso una lampada / *I turned on a lamp* | 21 |
|  | martello | hammer | Ho usato un martello / *I used a hammer* | 20 |
|  | scopa | broom | Ho sistemato la scopa / *I fixed the broom* | 22 |
|  | bottiglia | bottle | Ho preso una bottiglia / *I got a bottle* | 23 |
|  | coltello | knife | Ho affilato un coltello / *I sharpened a knife* | 23 |
|  | trapano | drill | Ho utilizzato il trapano / *I used the drill* | 24 |
|  | ombrello | umbrella | Ho comprato l'ombrello / *I bought the umbrella* | 22 |
|  | forchetta | fork | Ho lavato la forchetta / *I washed the fork* | 22 |
|  | matita | pencil | Ho temperato la matita / *I sharpened the pencil* | 22 |
|  | pennello | brush | Ho lavato un pennello / *I washed the brush* | 21 |
|  |  |  |  |  |
| Animals | cane | dog | Ho accarezzato il cane / *I patted the dog* | 23 |
|  | leone | lion | Ho fotografato il leone / *I photographed the lion* | 24 |
|  | maiale | pig | Ho trovato un maiale / *I found a pig* | 20 |
|  | cammello | camel | Ho visto un cammello / *I saw a camel* | 20 |
|  | pecora | sheep | Ho notato una pecora / *I noticed a sheep* | 20 |
|  | mucca | cow | Ho osservato la mucca / *I observed the cow* | 21 |
|  | piccione | pigeon | Ho evitato un piccione / *I avoided a pigeon* | 22 |
|  | gallina | hen | Ho cercato la gallina / *I looked for the hen* | 22 |
|  | pappagallo | parrott | Ho sentito il pappagallo / *I heard the parrot* | 24 |
|  | insetto | insect | Ho catturato un insetto / *I caught an insect* | 23 |

**Tab. 2 List of abstract sentences. Abbreviation category: PSTQ (physical-spatio-temporal-quantitative), PS (philosophical-spiritual), EMSS (Emotional-Social)**

| **Category** | **Italian Word** | **English Word** | **Abstract Sentence** | | **Lenght** | |
| --- | --- | --- | --- | --- | --- | --- |
| PSTQ | accelerazione | acceleration | Ho stimato l'accelerazione / *I estimaed the acceleration* | | | 26 |
|  | inizio | start | Ho sperato in un inizio / *I hoped for a start* | | | 23 |
|  | schema | scheme | Ho creato uno schema / *I created a scheme* | | | 21 |
|  | area | area | Ho delimitato l'area / *I demarced the area* | | | 20 |
|  | numero | number | Ho calcolato il numero / *I calculated the number* | | | 22 |
|  | risultato | result | Ho ottenuto il risultato / *I got the result* | | | 24 |
|  | punizione | punishment | Ho subito una punizione / *I suffered punishment* | | | 23 |
|  | rimedio | remedy | Ho trovato un rimedio / *I found a remedy* | | | 22 |
|  | sforzo | attempt | Ho premiato lo sforzo / *I rewarded the attempt* | | | 21 |
|  | denaro | money | Ho pensato al denaro / *I thought about money* | | | 20 |
|  |  |  |  | | |  |
| PS | morale | moral | Ho seguito la morale / *I followed the moral* | | | 20 |
|  | descrizione | description | Ho fatto una descrizione / *I made a description* | | | 25 |
|  | motivo | reason | Ho compreso il motivo / *I understand the reason* | | | 21 |
|  | salvezza | salvation | Ho creduto nella salvezza / *I believed in salvation* | | | 25 |
|  | destino | destiny | Ho pensato al destino / *I thought about destiny* | | | 21 |
|  | paradiso | paradise | Ho sognato il paradiso / *I dreamed of paradise* | | | 22 |
|  | enigma | enigma | Ho risolto un enigma / *I solved an enigma* | | | 21 |
|  | peccato | sin | Ho commesso un peccato / *I committed a sin* | | | 22 |
|  | giudizio | judgement | Ho espresso un giudizio / *I made a judgment* | | | 23 |
|  | logica | logic | Ho cercato una logica / *I looked for a logic* | | | 22 |
|  |  |  |  | | |  |
| EMSS | calma | calm | Ho percepito la calma / *I felt the calm* | 21 | | |
|  | gioia | joy | Ho manifestato gioia / *I expressed joy* | 20 | | |
|  | amicizia | friendship | Ho stretto un'amicizia / *I made a friendship* | 22 | | |
|  | conflitto | conflict | Ho evitato un conflitto / *I avoided a conflict* | 23 | | |
|  | gentilezza | kindness | Ho fatto una gentilezza / *I did a kindness* | 23 | | |
|  | vendetta | revenge | Ho gustato la vendetta / *I tasted the revenge* | 22 | | |
|  | ansia | anxiety | Ho controllato l'ansia / *I managed the anxiety* | 22 | | |
|  | vergogna | shame | Ho provato vergogna / *I felt ashemed* | 20 | | |
|  | simpatia | sympathy | Ho nutrito una simpatia / *I had a sympathy* | 23 | | |
|  | paura | fear | Ho affrontato la paura / *I faced the fear* | 23 | | |
