# Supplementary material for: Abstract and concrete concepts in conversation
Source: Sci Rep. 2022 Oct 20;12:17572. doi: 10.1038/s41598-022-20785-5 (PMC9584910; doi:10.1038/s41598-022-20785-5)

**Figure 1.** Polar plots showing the percentage of 1^st^ person perspective (**A**) for sentences including tool (*dark turquoise*), animal (*magenta*), food (*medium orchid*) concepts; the 1st or 2^nd^ person (**B**) and 1^st^ & 2^nd^ person (**C**) perspective for sentences including PS, philosophical-spiritual (*magenta*), EMSS, emotional-social (*medium orchid*), and PSTQ, physical-spatio-temporal-quantitative (*dark turquoise*) concepts. Polar plots showing the percentage of 3^rd^ person perspective (**D**) and general statement variables (**E**) and the row frequency count of the number of evoked contexts variable (**F**) for sentence including animals (*blue*), tools (*light green*), food (*orange*), PS, philosophical-spiritual (*aqua*), EMSS, emotional-social (*gold*), and PSTQ, physical-spatio-temporal-quantitative (*violet*) concepts


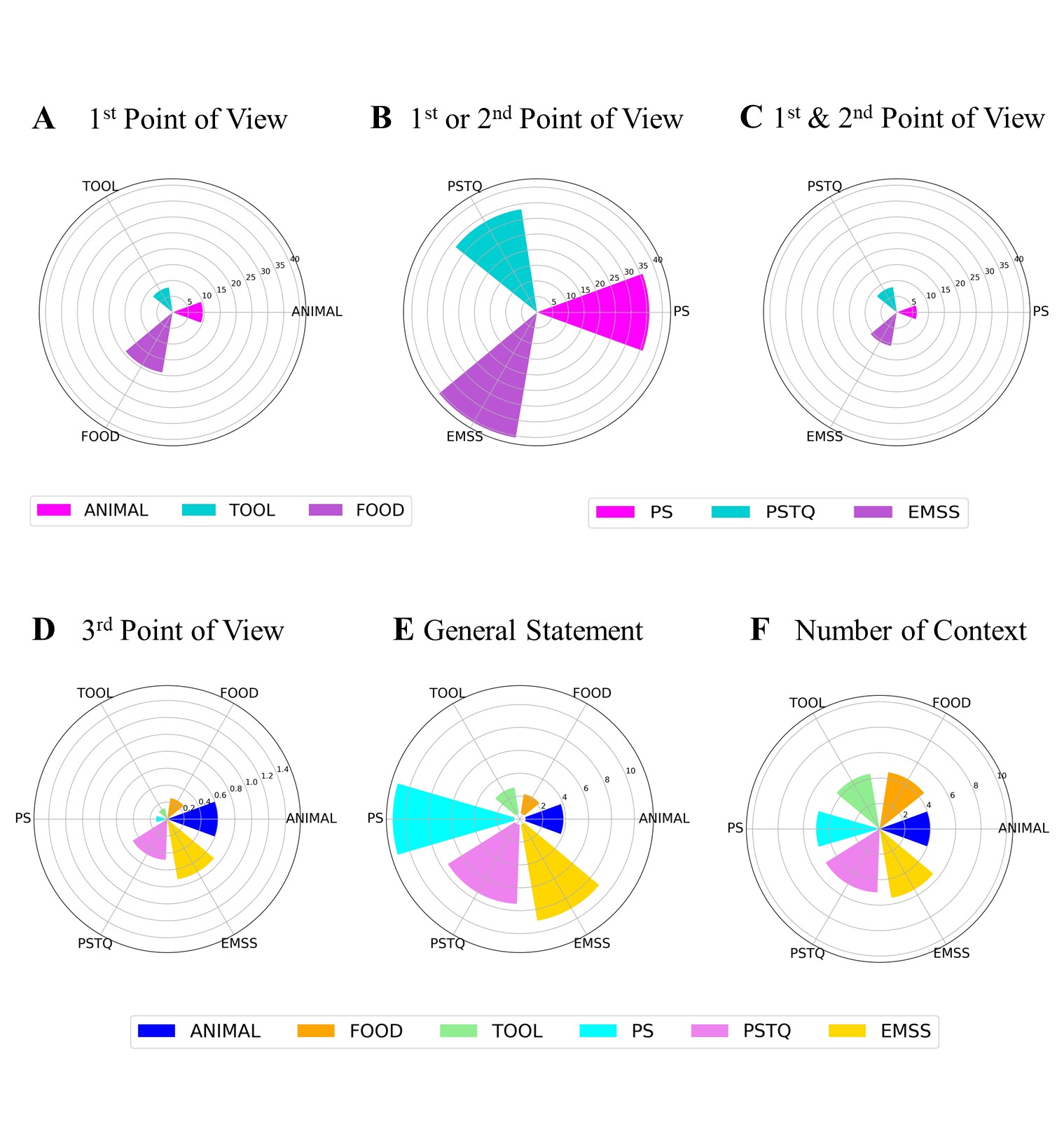


**Figure 2.** Polar plots for sentences including animals (**A**), tools (**B**), food (**C**), PS philosophical-spiritual (**D**), EMSS emotional-social (**E**), and PSTQ, physical-spatio-temporal-quantitative (**F**) concepts, showing the percentage of thematic relations i.e., spatial (*slate blue*), temporal (*cornflowerblue*), events (*tomato*). Polar plots showing the percentage of concrete action (**G**) and abstract action (**H**) variables for sentences including animals (*blue*), tools (*light* green), food (*orange*), PS, philosophical-spiritual (*aqua*), EMSS, emotional-social *gold*), and PSTQ, physical-spatio-temporal-quantitative (*violet*) concepts.


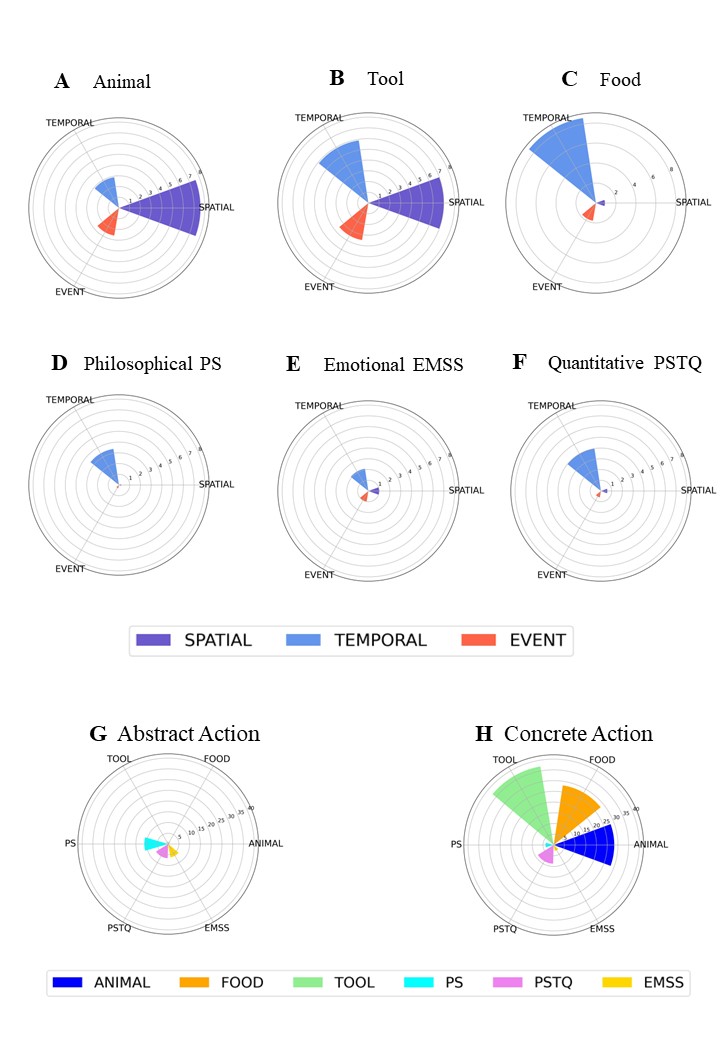


**Figure 3.** Polar plots for sentences including animals (**A**), tools (**B**), food (**C**), PS, philosophical-spiritual (**D**), EMSS, emotional-social (**E**), and PSTQ, physical-spatio-temporal-quantitative (**F**) concepts, showing the percentage of other dimensions,i.e., associations (*gold*), subordinates (*medium sea green*), non-perceptual evaluations (*medium purple*).


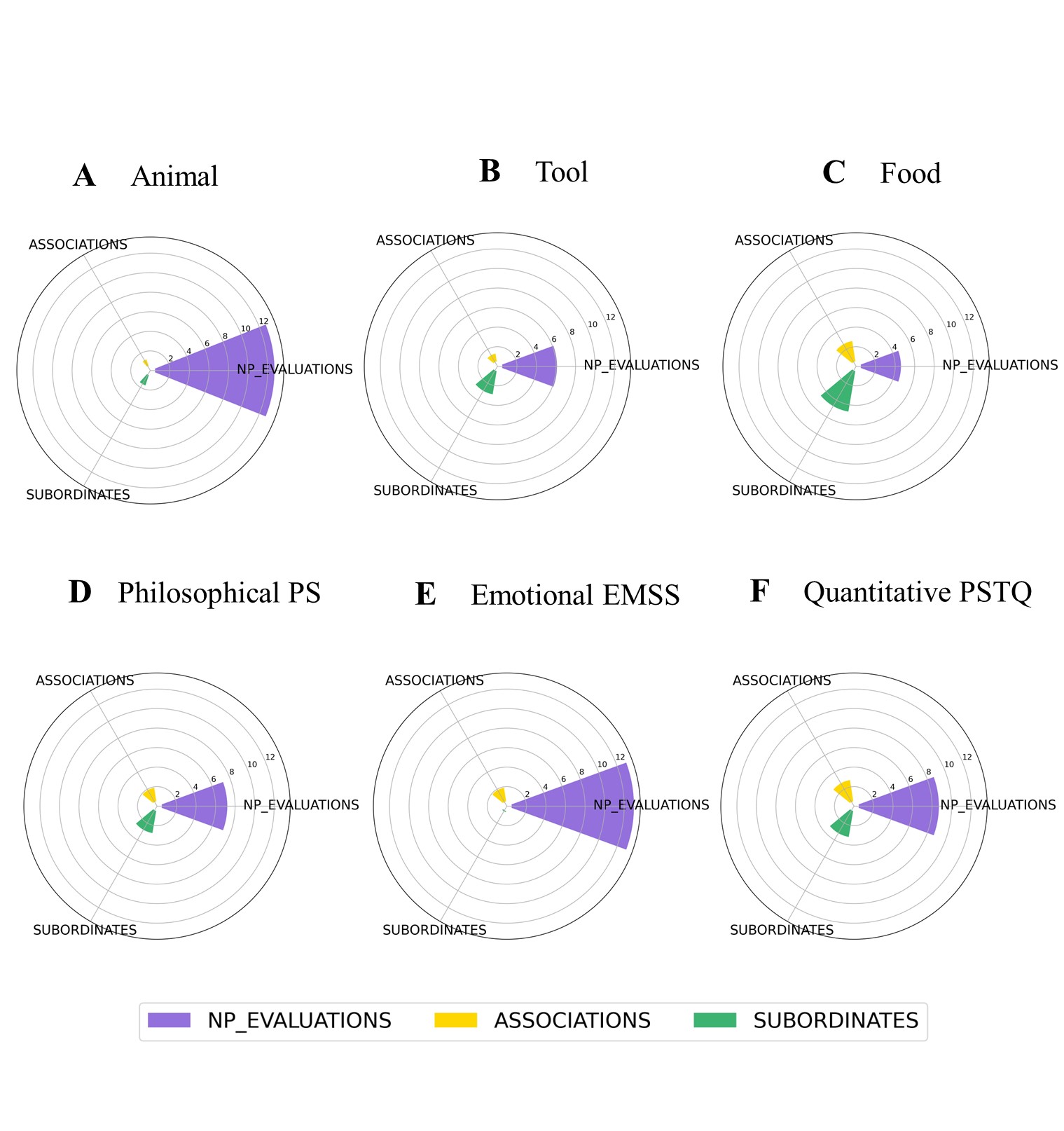

Supplement: Supplementary file 2 — Supplementary Information 2. [file 41598_2022_20785_MOESM2_ESM.docx]
