# Supplementary material for: Abstract and concrete concepts in conversation
Source: Sci Rep. 2022 Oct 20;12:17572. doi: 10.1038/s41598-022-20785-5 (PMC9584910; doi:10.1038/s41598-022-20785-5)
Supplement: Supplementary file 3 — Supplementary Information 3. [file 41598_2022_20785_MOESM3_ESM.docx]

**Model Specifications**

Estimates and 95% posterior credibility intervals (PCIs) for all the 37 models, according to Bayes Factor (BF) and model selection. For each variables, Standard Error, Rhat, Bulk_ESS and Tail_ESS values, related to model convergence and suitability of effective sample size are reported in separate table.

# Abstract Action

| Variables | Estimate | Std..Error | Lower.95. | Upper.95. | Rhat | Bulk_ESS | Tail_ESS |
| --- | --- | --- | --- | --- | --- | --- | --- |
| Intercept | -5.43 | 0.48 | -6.47 | -4.57 | 1.00 | 7582 | 8889 |
| SubtypeEMSS | 2.72 | 0.49 | 1.85 | 3.79 | 1.00 | 7875 | 8869 |
| Subtypefood | 0.75 | 0.57 | -0.34 | 1.93 | 1.00 | 9330 | 10930 |
| SubtypePS | 3.35 | 0.48 | 2.48 | 4.40 | 1.00 | 7764 | 8976 |
| SubtypePSQT | 2.78 | 0.49 | 1.90 | 3.83 | 1.00 | 7791 | 8890 |
| Subtypetool | 0.95 | 0.56 | -0.09 | 2.10 | 1.00 | 9052 | 10035 |
| sd(Intercept) ss_ID | 0.76 | 0.11 | 0.56 | 0.99 | 1.00 | 9600 | 14765 |
| WAIC | 1738.51 | 80.65 |  |  |  |  |  |

# Concrete Action

| Variables | Estimate | Std..Error | Lower.95. | Upper.95. | Rhat | Bulk_ESS | Tail_ESS |
| --- | --- | --- | --- | --- | --- | --- | --- |
| Intercept | -0.96 | 0.1 | -1.15 | -0.77 | 1.00 | 12609 | 16421 |
| SubtypeEMSS | -2.83 | 0.24 | -3.33 | -2.37 | 1.00 | 24355 | 18395 |
| Subtypefood | -0.01 | 0.11 | -0.23 | 0.21 | 1.00 | 20142 | 18775 |
| SubtypePS | -2.44 | 0.21 | -2.86 | -2.04 | 1.00 | 25410 | 18569 |
| SubtypePSQT | -1.47 | 0.15 | -1.76 | -1.18 | 1.00 | 24486 | 18722 |
| Subtypetool | 0.4 | 0.11 | 0.19 | 0.62 | 1.00 | 20638 | 18856 |
| sd(Intercept) ss_ID | 0.49 | 0.07 | 0.36 | 0.63 | 1.00 | 10019 | 15686 |
| WAIC | 3822.83 | 77.83 |  |  |  |  |  |

# Associations

| Variables | Estimate | Std..Error | Lower.95. | Upper.95. | Rhat | Bulk_ESS | Tail_ESS |
| --- | --- | --- | --- | --- | --- | --- | --- |
| Intercept | -5.07 | 0.4 | -5.92 | -4.33 | 1.00 | 9015 | 10288 |
| SubtypeEMSS | 0.74 | 0.45 | -0.11 | 1.66 | 1.00 | 10665 | 11991 |
| Subtypefood | 1.18 | 0.43 | 0.39 | 2.05 | 1.00 | 10097 | 11976 |
| SubtypePS | 0.74 | 0.45 | -0.10 | 1.66 | 1.00 | 10620 | 12336 |
| SubtypePSQT | 1.26 | 0.42 | 0.49 | 2.13 | 1.00 | 9438 | 12358 |
| Subtypetool | 0.13 | 0.51 | -0.86 | 1.13 | 1.00 | 12266 | 14358 |
| sd(Intercept) ss_ID | 0.97 | 0.19 | 0.64 | 1.37 | 1.00 | 8206 | 12913 |
| WAIC | 919.64 | 73.33 |  |  |  |  |  |

# Belief

| Variables | Estimate | Std..Error | Lower.95. | Upper.95. | Rhat | Bulk_ESS | Tail_ESS |
| --- | --- | --- | --- | --- | --- | --- | --- |
| Intercept | -3.1 | 0.18 | -3.46 | -2.76 | 1.00 | 9015 | 10288 |
| SubtypeEMSS | 1.25 | 0.19 | 0.88 | 1.63 | 1.00 | 10665 | 11991 |
| Subtypefood | -0.37 | 0.25 | -0.86 | 0.11 | 1.00 | 10097 | 11976 |
| SubtypePS | 1.35 | 0.19 | 0.98 | 1.73 | 1.00 | 10620 | 12336 |
| SubtypePSQT | 0.98 | 0.19 | 0.61 | 1.37 | 1.00 | 9438 | 12358 |
| Subtypetool | 0.32 | 0.21 | -0.09 | 0.74 | 1.00 | 12266 | 14358 |
| sd(Intercept) ss_ID | 0.65 | 0.09 | 0.49 | 0.85 | 1.00 | 8136 | 14287 |
| WAIC | 2885.72 | 88.14 |  |  |  |  |  |

# Emotions

| Variables | Estimate | Std..Error | Lower.95. | Upper.95. | Rhat | Bulk_ESS | Tail_ESS |
| --- | --- | --- | --- | --- | --- | --- | --- |
| Intercept | -3.2 | 0.19 | -3.58 | -2.86 | 1.00 | 17320 | 17159 |
| SubtypeEMSS | 1.18 | 0.21 | 0.78 | 1.60 | 1.00 | 18032 | 17972 |
| Subtypefood | -0.98 | 0.34 | -1.67 | -0.34 | 1.00 | 25236 | 19508 |
| SubtypePS | -0.1 | 0.26 | -0.61 | 0.41 | 1.00 | 20196 | 19508 |
| SubtypePSQT | -0.03 | 0.26 | -0.54 | 0.46 | 1.00 | 18915 | 19004 |
| Subtypetool | -0.58 | 0.3 | -1.17 | -0.01 | 1.00 | 23083 | 18366 |
| sd(Intercept) ss_ID | 0.3 | 0.13 | 0.03 | 0.55 | 1.00 | 5949 | 6886 |
| WAIC | 1713.43 | 85.01 |  |  |  |  |  |

# Events

| Variables | Estimate | Std..Error | Lower.95. | Upper.95. | Rhat | Bulk_ESS | Tail_ESS |
| --- | --- | --- | --- | --- | --- | --- | --- |
| Intercept | -3.67 | 0.25 | -4.18 | -3.21 | 1.00 | 15065 | 17268 |
| SubtypeEMSS | -0.98 | 0.35 | -1.68 | -0.32 | 1.00 | 23062 | 17160 |
| Subtypefood | -0.36 | 0.29 | -0.94 | 0.19 | 1.00 | 23206 | 19234 |
| SubtypePS | -2.26 | 0.56 | -3.45 | -1.27 | 1.00 | 26010 | 15315 |
| SubtypePSQT | -1.5 | 0.41 | -2.35 | -0.75 | 1.00 | 25552 | 18139 |
| Subtypetool | 0.34 | 0.25 | -0.14 | 0.83 | 1.00 | 21010 | 19483 |
| sd(Intercept) ss_ID | 1.06 | 0.17 | 0.77 | 1.43 | 1.00 | 8182 | 13521 |
| WAIC | 1018.06 | 71.88 |  |  |  |  |  |

# General Statement

| Variables | Estimate | Std..Error | Lower.95. | Upper.95. | Rhat | Bulk_ESS | Tail_ESS |
| --- | --- | --- | --- | --- | --- | --- | --- |
| Intercept | -3.41 | 0.21 | -3.82 | -3.01 | 1.00 | 13451 | 14418 |
| SubtypeEMSS | 1.02 | 0.22 | 0.60 | 1.45 | 1.00 | 15356 | 17586 |
| Subtypefood | -0.71 | 0.31 | -1.33 | -0.12 | 1.00 | 22161 | 18141 |
| SubtypePS | 1.27 | 0.21 | 0.86 | 1.69 | 1.00 | 15147 | 15587 |
| SubtypePSQT | 0.79 | 0.22 | 0.35 | 1.23 | 1.00 | 16316 | 17811 |
| Subtypetool | -0.39 | 0.28 | -0.94 | 0.16 | 1.00 | 19599 | 18453 |
| sd(Intercept) ss_ID | 0.78 | 0.11 | 0.58 | 1.02 | 1.00 | 8734 | 14246 |
| WAIC | 2162.16 | 85.88 |  |  |  |  |  |

# Hearing

| Variables | Estimate | Std..Error | Lower.95. | Upper.95. | Rhat | Bulk_ESS | Tail_ESS |
| --- | --- | --- | --- | --- | --- | --- | --- |
| Intercept | -8.59 | 1.21 | -11.40 | -6.70 | 1.00 | 15272 | 10793 |
| TypeConceptconcrete | 2.98 | 1.15 | 3.3 | 280.09 | 1.00 | 21999 | 11721 |
| sd(Intercept) ss_ID | 0.98 | 0.51 | 0.08 | 2.05 | 1.00 | 5822 | 7466 |
| WAIC | 201.74 | 43.01 |  |  |  |  |  |

# How

| Variables | Estimate | Std..Error | Lower.95. | Upper.95. | Rhat | Bulk_ESS | Tail_ESS |
| --- | --- | --- | --- | --- | --- | --- | --- |
| Intercept | -2.95 | 0.17 | -3.29 | -2.63 | 1.00 | 11989 | 14639 |
| SubtypeEMSS | 0.55 | 0.2 | 0.16 | 0.95 | 1.00 | 14398 | 17128 |
| Subtypefood | 1.41 | 0.18 | 1.06 | 1.78 | 1.00 | 13402 | 15593 |
| SubtypePS | 0.88 | 0.19 | 0.50 | 1.26 | 1.00 | 13940 | 17628 |
| SubtypePSQT | 0.43 | 0.2 | 0.03 | 0.84 | 1.00 | 14837 | 18029 |
| Subtypetool | -0.62 | 0.26 | -1.15 | -0.13 | 1.00 | 18446 | 19219 |
| sd(Intercept) ss_ID | 0.43 | 0.08 | 0.27 | 0.59 | 1.00 | 9676 | 11729 |
| WAIC | 2809.81 | 89.04 |  |  |  |  |  |

# Interoception

| Variables | Estimate | Std..Error | Lower.95. | Upper.95. | Rhat | Bulk_ESS | Tail_ESS |
| --- | --- | --- | --- | --- | --- | --- | --- |
| Intercept | -7.25 | 1.22 | -10.15 | -5.40 | 1.00 | 8389 | 6942 |
| SubtypeEMSS | 0.94 | 1.44 | -1.65 | 4.08 | 1.00 | 9058 | 7464 |
| Subtypefood | 3.24 | 1.24 | 1.33 | 6.15 | 1.00 | 8539 | 6988 |
| SubtypePS | -26.04 | 47.67 | -133.69 | 0.42 | 1.00 | 3668 | 1858 |
| SubtypePSQT | -0.01 | 1.73 | -3.48 | 3.43 | 1.00 | 9344 | 8260 |
| Subtypetool | 1.43 | 1.36 | -0.91 | 4.50 | 1.00 | 8543 | 7148 |
| sd(Intercept) ss_ID | 0.35 | 0.26 | 0.01 | 0.97 | 1.00 | 8240 | 9951 |
| WAIC | 269.42 | 48.15 |  |  |  |  |  |

# Introspection

| Variables | Estimate | Std..Error | Lower.95. | Upper.95. | Rhat | Bulk_ESS | Tail_ESS |
| --- | --- | --- | --- | --- | --- | --- | --- |
| Intercept | -4.44 | 0.4 | -5.22 | -3.64 | 1.00 | 14011 | 15884 |
| SubtypeEMSS | 0.37 | 0.39 | -0.38 | 1.13 | 1.00 | 16942 | 16943 |
| Subtypefood | -1.5 | 0.7 | -3.01 | -0.25 | 1.00 | 23449 | 15107 |
| SubtypePS | 0.71 | 0.36 | 0.02 | 1.44 | 1.00 | 16276 | 15664 |
| SubtypePSQT | 0.08 | 0.41 | -0.73 | 0.89 | 1.00 | 18243 | 16341 |
| Subtypetool | -0.31 | 0.46 | 0.29 | 1.79 | 1.00 | 18493 | 17330 |
| sd(Intercept) ss_ID | 0.93 | 0.18 | 0.63 | 1.31 | 1.00 | 9994 | 15286 |
| WAIC | 747.76 | 69.47 |  |  |  |  |  |

# Material

| Variables | Estimate | Std..Error | Lower.95. | Upper.95. | Rhat | Bulk_ESS | Tail_ESS |  |
| --- | --- | --- | --- | --- | --- | --- | --- | --- |
| Intercept | -15.6 | 10.15 | -43.57 | -7.64 | 1.00 | 5340 | 2866 |  |
| TypeConceptconcrete | 12.28 | 10.15 | 4.31 | 40.24 | 1.00 | 5333 | 2868 |  |
| sd(Intercept) ss_ID | 0.65 | 0.18 | 0.29 | 1.01 | 1.00 | 7613 | 6859 |  |
| WAIC | 827.97 | 62.17 |  |  |  |  |  |  |

# Metacognition

| Variables | Estimate | Std..Error | Lower.95. | Upper.95. | Rhat | Bulk_ESS | Tail_ESS |  |
| --- | --- | --- | --- | --- | --- | --- | --- | --- |
| Intercept | -49.87 | 84.28 | -229.70 | -7.79 | 1.00 | 2321 | 1195 |  |
| SubtypeEMSS | 42.26 | 84.29 | -0.13 | 222.15 | 1.00 | 2337 | 1193 |  |
| Subtypefood | 42.26 | 84.29 | -0.10 | 221.78 | 1.00 | 2332 | 1194 |  |
| SubtypePS | 43.72 | 84.27 | 1.62 | 223.35 | 1.00 | 2338 | 1196 |  |
| SubtypePSQT | 45.56 | 84.28 | 3.45 | 225.30 | 1.00 | 2314 | 1196 |  |
| Subtypetool | 1.08 | 85.7 | -144.55 | 149 | 1.00 | 3407 | 1599 |  |
| sd(Intercept) ss_ID | 0.85 | 0.45 | 0.07 | 1.8 | 1.00 | 5702 | 9810 |  |
| WAIC | 236.14 | 44.08 |  |  |  |  |  |  |

# Number of Questions

| Variables | Estimate | Std..Error | Lower.95. | Upper.95. | Rhat | Bulk_ESS | Tail_ESS |
| --- | --- | --- | --- | --- | --- | --- | --- |
| Intercept | -0.57 | 0.06 | -0.68 | -0.46 | 1.00 | 10242 | 14359 |
| SubtypeEMSS | -0.41 | 0.07 | -0.55 | -0.27 | 1.00 | 18693 | 18308 |
| Subtypefood | -0.24 | 0.07 | -0.37 | -0.10 | 1.00 | 17993 | 18261 |
| SubtypePS | -0.04 | 0.07 | -0.17 | 0.09 | 1.00 | 16516 | 18832 |
| SubtypePSQT | -0.12 | 0.07 | -0.25 | 0.01 | 1.00 | 17626 | 18119 |
| Subtypetool | -0.33 | 0.07 | -0.47 | -0.19 | 1.00 | 18700 | 18975 |
| sd(Intercept) ss_ID | 0.28 | 0.03 | 0.21 | 0.35 | 1.00 | 9364 | 14695 |
| WAIC | 7964.65 | 59.69 |  |  |  |  |  |

# Non-Perceptual Evaluation

| Variables | Estimate | Std..Error | Lower.95. | Upper.95. | Rhat | Bulk_ESS | Tail_ESS |
| --- | --- | --- | --- | --- | --- | --- | --- |
| Intercept | -1.97 | 0.12 | -2.22 | -1.75 | 1.00 | 14161 | 15726 |
| SubtypeEMSS | 0.02 | 0.15 | -0.27 | 0.32 | 1.00 | 17766 | 18212 |
| Subtypefood | -1.21 | 0.2 | -1.62 | -0.81 | 1.00 | 20985 | 17783 |
| SubtypePS | -0.68 | 0.17 | -1.02 | -0.34 | 1.00 | 20316 | 18696 |
| SubtypePSQT | -0.45 | 0.17 | -0.78 | -0.12 | 1.00 | 19918 | 18424 |
| Subtypetool | -0.87 | 0.18 | -1.23 | -0.51 | 1.00 | 19740 | 17298 |
| sd(Intercept) ss_ID | 0.44 | 0.08 | 0.28 | 0.61 | 1.00 | 8764 | 11353 |
| WAIC | 2763.94 | 90.32 |  |  |  |  |  |

# Number of Target Word Repetition

| Variables | Estimate | Std..Error | Lower.95. | Upper.95. | Rhat | Bulk_ESS | Tail_ESS |
| --- | --- | --- | --- | --- | --- | --- | --- |
| Intercept | -2.73 | 0.19 | -3.11 | -2.38 | 1.00 | 11842 | 16057 |
| SubtypeEMSS | -0.96 | 0.23 | .1.42 | -0.51 | 1.00 | 25913 | 19589 |
| Subtypefood | -0.14 | 0.19 | -0.52 | 0.23 | 1.00 | 23113 | 19010 |
| SubtypePS | -0.22 | 0.19 | -0.60 | 0.16 | 1.00 | 22948 | 19651 |
| SubtypePSQT | -0.96 | 0.23 | -1.43 | -0.52 | 1.00 | 25665 | 18672 |
| Subtypetool | -1.95 | 0.32 | -2.61 | -1.34 | 1.00 | 29157 | 18857 |
| sd(Intercept) ss_ID | 1.04 | 0.14 | 0.79 | 1.34 | 1.00 | 7042 | 11371 |
| WAIC | 1826.76 | 82.83 |  |  |  |  |  |

# Number of Context

| Variables | Estimate | Std..Error | Lower.95. | Upper.95. | Rhat | Bulk_ESS | Tail_ESS |
| --- | --- | --- | --- | --- | --- | --- | --- |
| Intercept | 1.64 | 0.08 | 1.48 | 1.79 | 1.00 | 14147 | 12252 |
| TypeConceptconcrete | -0.19 | 0.12 | -0.42 | 0.05 | 1.00 | 12714 | 11961 |
| WAIC | 227.42 | 5.2 |  |  |  |  |  |

# Point of View

| Variables | Estimate | Std..Error | Lower.95. | Upper.95. | Rhat | Bulk_ESS | Tail_ESS |
| --- | --- | --- | --- | --- | --- | --- | --- |
| Intercept | -0.16 | 0.07 | -0.30 | -0.02 | 1.00 | 6164 | 10422 |
| TypeConceptconcrete | 0.07 | 0.06 | -0.05 | 0.18 | 1.00 | 29441 | 18203 |
| sd(Intercept) ss_ID | 0.51 | 0.06 | 0.41 | 0.63 | 1.00 | 6897 | 12187 |
| WAIC | 6437.7 | 28.32 |  |  |  |  |  |

# 1^st^ or 2^nd^ Point of View

| Variables | Estimate | Std..Error | Lower.95. | Upper.95. | Rhat | Bulk_ESS | Tail_ESS |
| --- | --- | --- | --- | --- | --- | --- | --- |
| Intercept | -0.4 | 0.08 | -0.57 | -0.24 | 1.00 | 24069 | 18220 |
| SubtypePS | -0.2 | 0.1 | -0.41 | 0.00 | 1.00 | 34495 | 18057 |
| SubtypePSQT | -0.32 | 0.11 | -0.52 | -0.11 | 1.00 | 34828 | 18186 |
| sd(Intercept) ss_ID | 0.37 | 0.07 | 0.24 | 0.5 | 1.00 | 10165 | 13909 |
| WAIC | 3117.23 | 28.53 |  |  |  |  |  |

# 1^st^ & 2^nd^ Point of View

| Variables | Estimate | Std..Error | Lower.95. | Upper.95. | Rhat | Bulk_ESS | Tail_ESS |
| --- | --- | --- | --- | --- | --- | --- | --- |
| Intercept | -0.97 | 0.07 | -1.11 | -0.84 | 1.00 | 11404 | 15338 |
| TypeConceptconcrete | -0.08 | 0.07 | -0.21 | 0.05 | 1.00 | 33348 | 17251 |
| sd(Intercept) ss_ID | 0.44 | 0.05 | 0.34 | 0.55 | 1.00 | 9269 | 15337 |
| WAIC | 5538.99 | 61.97 |  |  |  |  |  |

# 2^nd^ Point of View

| Variables | Estimate | Std..Error | Lower.95. | Upper.95. | Rhat | Bulk_ESS | Tail_ESS |
| --- | --- | --- | --- | --- | --- | --- | --- |
| Intercept | -2.18 | 0.14 | -2.46 | -1.91 | 1.00 | 20716 | 17911 |
| SubtypePS | -0.62 | 0.18 | -0.98 | -0.27 | 1.00 | 30992 | 18247 |
| SubtypePSQT | -0.35 | 0.17 | -0.68 | -0.02 | 1.00 | 30901 | 18583 |
| sd(Intercept) ss_ID | 0.64 | 0.12 | 0.42 | 0.89 | 1.00 | 9557 | 14754 |
| WAIC | 1412.97 | 63.86 |  |  |  |  |  |

# 1^st^ Point of View

| Variables | Estimate | Std..Error | Lower.95. | Upper.95. | Rhat | Bulk_ESS | Tail_ESS |
| --- | --- | --- | --- | --- | --- | --- | --- |
| Intercept | -2.32 | 0.16 | -2.64 | -2.02 | 1.00 | 12706 | 15133 |
| Subtypefood | 0.85 | 0.15 | 0.56 | 1.14 | 1.00 | 26705 | 18717 |
| Subtypetool | -0.22 | 0.17 | -0.55 | 0.12 | 1.00 | 27567 | 18895 |
| sd(Intercept) ss_ID | 0.92 | 0.12 | 0.7 | 1.16 | 1.00 | 7051 | 10443 |
| WAIC | 1778.41 | 59.93 |  |  |  |  |  |

# 3^rd^ Point of View

| Variables | Estimate | Std..Error | Lower.95. | Upper.95. | Rhat | Bulk_ESS | Tail_ESS |
| --- | --- | --- | --- | --- | --- | --- | --- |
| Intercept | -5.23 | 0.5 | -6.31 | -4.34 | 1.00 | 16387 | 14453 |
| SubtypeEMSS | 0.2 | 0.64 | -1.05 | 1.48 | 1.00 | 16630 | 16584 |
| Subtypefood | -1.05 | 0.92 | -3.02 | 0.58 | 1.00 | 17984 | 15518 |
| SubtypePS | -1.98 | 1.31 | -5.01 | 0.10 | 1.00 | 15239 | 11369 |
| SubtypePSQT | -0.24 | 0.71 | -1.67 | 1.12 | 1.00 | 17909 | 17204 |
| Subtypetool | -1.98 | 1.3 | -5.05 | 0.09 | 1.00 | 14812 | 10561 |
| sd(Intercept) ss_ID | 0.36 | 0.3 | 0.01 | 1.11 | 1.00 | 10192 | 10743 |
| WAIC | 257.06 | 50.57 |  |  |  |  |  |

# Smell

| Variables | Estimate | Std..Error | Lower.95. | Upper.95. | Rhat | Bulk_ESS | Tail_ESS |
| --- | --- | --- | --- | --- | --- | --- | --- |
| Intercept | -18.16 | 14.73 | -57.63 | -7.85 | 1.00 | 3545 | 1781 |
| TypeConceptconcrete | 11.05 | 14.64 | 0.93 | 50.24 | 1.00 | 3561 | 1763 |
| sd(Intercept) ss_ID | 0.96 | 0.78 | 0.04 | 2.88 | 1.00 | 8394 | 9937 |
| WAIC | 61.4 | 26.38 |  |  |  |  |  |

# Spatial

| Variables | Estimate | Std..Error | Lower.95. | Upper.95. | Rhat | Bulk_ESS | Tail_ESS |
| --- | --- | --- | --- | --- | --- | --- | --- |
| Intercept | -2.5 | 0.17 | -2.85 | -2.19 | 1.00 | 18728 | 18881 |
| SubtypeEMSS | -2.19 | 0.35 | -2.92 | -1.54 | 1.00 | 30327 | 15989 |
| Subtypefood | -2.31 | 0.37 | -3.07 | -1.63 | 1.00 | 31037 | 17629 |
| SubtypePS | -4 | 0.79 | -5.81 | -2.71 | 1.00 | 21934 | 12795 |
| SubtypePSQT | -2.74 | 0.44 | -3.68 | -1.94 | 1.00 | 31855 | 16885 |
| Subtypetool | -0.1 | 0.18 | -0.45 | 0.25 | 1.00 | 30111 | 19215 |
| sd(Intercept) ss_ID | 0.8 | 0.14 | 0.54 | 1.11 | 1.00 | 8823 | 14809 |
| WAIC | 1243.08 | 73.1 |  |  |  |  |  |

# Sub-Specification

| Variables | Estimate | Std..Error | Lower.95. | Upper.95. | Rhat | Bulk_ESS | Tail_ESS |
| --- | --- | --- | --- | --- | --- | --- | --- |
| Intercept | -4.53 | 0.33 | -5.22 | -3.93 | 1.00 | 11435 | 13214 |
| SubtypeEMSS | -2.82 | 1.25 | -5.71 | 0.41 | 1.00 | 30327 | 15989 |
| Subtypefood | 1.38 | 0.35 | 0.71 | 2.10 | 1.00 | 13067 | 14145 |
| SubtypePS | 0.74 | 0.38 | 0.01 | 1.49 | 1.00 | 13163 | 15293 |
| SubtypePSQT | 0.92 | 0.37 | 0.22 | 1.68 | 1.00 | 12639 | 15006 |
| Subtypetool | 0.79 | 0.37 | 0.07 | 1.55 | 1.00 | 13279 | 14489 |
| sd(Intercept) ss_ID | 0.69 | 0.17 | 0.37 | 1.02 | 1.00 | 7871 | 9262 |
| WAIC | 1083.07 | 75.82 |  |  |  |  |  |

# Taste

| Variables | Estimate | Std..Error | Lower.95. | Upper.95. | Rhat | Bulk_ESS | Tail_ESS |
| --- | --- | --- | --- | --- | --- | --- | --- |
| Intercept | -15.54 | 12.22 | -44.08 | -7.39 | 1.00 | 4210 | 1849 |
| TypeConceptconcrete | 13.33 | 12.22 | 5.18 | 41.84 | 1.00 | 4209 | 1849 |
| sd(Intercept) ss_ID | 0.12 | 0.08 | 0 | 0.31 | 1.00 | 10033 | 11368 |
| WAIC | 1560.23 | 68.58 |  |  |  |  |  |

# Temporal

| Variables | Estimate | Std..Error | Lower.95. | Upper.95. | Rhat | Bulk_ESS | Tail_ESS |
| --- | --- | --- | --- | --- | --- | --- | --- |
| Intercept | -3.53 | 0.21 | -3.97 | -3.13 | 1.00 | 16781 | 14959 |
| SubtypeEMSS | -0.33 | 0.31 | -0.95 | 0.27 | 1.00 | 21307 | 18429 |
| Subtypefood | 1.15 | 0.24 | 0.70 | 1.63 | 1.00 | 18012 | 17583 |
| SubtypePS | 0.15 | 0.28 | -0.39 | 0.70 | 1.00 | 19900 | 18268 |
| SubtypePSQT | 0.35 | 0.26 | -0.17 | 0.87 | 1.00 | 19399 | 17879 |
| Subtypetool | 0.75 | 0.25 | 0.26 | 1.24 | 1.00 | 18721 | 18437 |
| sd(Intercept) ss_ID | 0.51 | 0.11 | 0.3 | 0.73 | 1.00 | 9510 | 11254 |
| WAIC | 1832.5 | 87.12 |  |  |  |  |  |

# Touch

| Variables | Estimate | Std..Error | Lower.95. | Upper.95. | Rhat | Bulk_ESS | Tail_ESS |
| --- | --- | --- | --- | --- | --- | --- | --- |
| Intercept | -4.94 | 0.53 | -6.13 | -4.06 | 1.00 | 16781 | 14959 |
| SubtypeEMSS | -48.04 | 91.6 | -242.46 | -3.04 | 1.00 | 3552 | 2042 |
| Subtypefood | -2.8 | 1.29 | -5.81 | -0.83 | 1.00 | 27518 | 12241 |
| SubtypePS | -46.78 | 78.45 | -243.00 | -3.03 | 1.00 | 3705 | 2600 |
| SubtypePSQT | -2.79 | 1.27 | -5.75 | -0.84 | 1.00 | 28847 | 14299 |
| Subtypetool | -0.12 | 0.47 | -1.04 | 0.81 | 1.00 | 38502 | 17492 |
| sd(Intercept) ss_ID | 1.01 | 0.48 | 0.1 | 2 | 1.00 | 4954 | 5636 |
| WAIC | 243.55 | 44.66 |  |  |  |  |  |

# Turn

| Variables | Estimate | Std..Error | Lower.95. | Upper.95. | Rhat | Bulk_ESS | Tail_ESS |
| --- | --- | --- | --- | --- | --- | --- | --- |
| Intercept | 0.34 | 0.1 | 0.15 | 0.54 | 1.00 | 6573 | 11059 |
| SubtypeEMSS | -0.79 | 0.11 | -1.00 | -0.58 | 1.00 | 14063 | 17841 |
| Subtypefood | -0.55 | 0.1 | -0.76 | -0.35 | 1.00 | 14959 | 16111 |
| SubtypePS | -0.01 | 0.1 | -0.21 | 0.19 | 1.00 | 14614 | 17128 |
| SubtypePSQT | -0.27 | 0.1 | -0.47 | -0.07 | 1.00 | 14934 | 17526 |
| Subtypetool | -0.69 | 0.1 | -0.89 | -0.48 | 1.00 | 14656 | 16549 |
| sd(Intercept) ss_ID | 0.6 | 0.06 | 0.49 | 0.72 | 1.00 | 6861 | 11690 |
| WAIC | 6271.21 | 38.69 |  |  |  |  |  |

# Uncertainty Expression

| Variables | Estimate | Std..Error | Lower.95. | Upper.95. | Rhat | Bulk_ESS | Tail_ESS |
| --- | --- | --- | --- | --- | --- | --- | --- |
| Intercept | -5.04 | 0.4 | -5.90 | -4.30 | 1.00 | 15238 | 17361 |
| SubtypeEMSS | -1.13 | 0.62 | -2.41 | 0 | 1.00 | 21723 | 16279 |
| Subtypefood | -28.14 | 51.03 | -125.36 | -2.83 | 1.00 | 2461 | 1184 |
| SubtypePS | 1 | 0.38 | 0.28 | 1.76 | 1.00 | 17755 | 16165 |
| SubtypePSQT | 0.1 | 0.44 | -0.76 | 0.95 | 1.00 | 18004 | 17895 |
| Subtypetool | -0.88 | 0.57 | -2.07 | 0.19 | 1.00 | 21598 | 17274 |
| sd(Intercept) ss_ID | 1.25 | 0.25 | 0.83 | 1.81 | 1.00 | 8030 | 13027 |
| WAIC | 545.07 | 59.32 |  |  |  |  |  |

#

# Vision

| Variables | Estimate | Std..Error | Lower.95. | Upper.95. | Rhat | Bulk_ESS | Tail_ESS |
| --- | --- | --- | --- | --- | --- | --- | --- |
| Intercept | -2.48 | 0.14 | -2.76 | -2.21 | 1.00 | 25535 | 19489 |
| SubtypeEMSS | -2.98 | 0.55 | -4.19 | -2.03 | 1.00 | 31956 | 16958 |
| Subtypefood | -0.31 | 0.2 | -0.69 | 0.08 | 1.00 | 33859 | 20171 |
| SubtypePS | -2.98 | 0.54 | -4.15 | -2.04 | 1.00 | 31036 | 15675 |
| SubtypePSQT | -2.1 | 0.36 | -2.86 | -1.43 | 1.00 | 38717 | 17630 |
| Subtypetool | -0.11 | 0.19 | -0.47 | 0.26 | 1.00 | 34034 | 20470 |
| sd(Intercept) ss_ID | 0.3 | 0.15 | 0.03 | 0.59 | 1.00 | 5096 | 6963 |
| WAIC | 1451.1 | 78.62 |  |  |  |  |  |

# What

| Variables | Estimate | Std..Error | Lower.95. | Upper.95. | Rhat | Bulk_ESS | Tail_ESS |
| --- | --- | --- | --- | --- | --- | --- | --- |
| Intercept | -2.22 | 0.13 | -2.49 | -1.97 | 1.00 | 12207 | 12648 |
| SubtypeEMSS | -1.14 | 0.22 | -1.58 | -0.72 | 1.00 | 19484 | 17906 |
| Subtypefood | -0.89 | 0.2 | -1.29 | -0.50 | 1.00 | 18745 | 16959 |
| SubtypePS | 0.93 | 0.14 | 0.64 | 1.21 | 1.00 | 15348 | 16976 |
| SubtypePSQT | 0.68 | 0.15 | 0.39 | 0.97 | 1.00 | 15345 | 16226 |
| Subtypetool | -0.48 | 0.18 | -0.84 | -0.12 | 1.00 | 17895 | 17240 |
| sd(Intercept) ss_ID | 0.56 | 0.08 | 0.42 | 0.72 | 1.00 | 8238 | 13751 |
| WAIC | 3106.13 | 87.02 |  |  |  |  |  |

# When

| Variables | Estimate | Std..Error | Lower.95. | Upper.95. | Rhat | Bulk_ESS | Tail_ESS |
| --- | --- | --- | --- | --- | --- | --- | --- |
| Intercept | -4.72 | 0.4 | -5.57 | -4.02 | 1.00 | 14911 | 18030 |
| SubtypeEMSS | -0.88 | 0.64 | -2.20 | 0.31 | 1.00 | 24293 | 17622 |
| Subtypefood | 0.4 | 0.45 | -0.47 | 1.29 | 1.00 | 22472 | 18265 |
| SubtypePS | -1.7 | 0.87 | -3.59 | -0.19 | 1.00 | 25067 | 14689 |
| SubtypePSQT | -25.26 | 35.3 | -111.75 | -2.50 | 1.00 | 3882 | 2057 |
| Subtypetool | -0.63 | 0.58 | -1.80 | 0.47 | 1.00 | 22777 | 18699 |
| sd(Intercept) ss_ID | 0.57 | 0.34 | 0.03 | 1.27 | 1.00 | 5400 | 7165 |
| WAIC | 381.06 | 54.96 |  |  |  |  |  |

# Where

| Variables | Estimate | Std..Error | Lower.95. | Upper.95. | Rhat | Bulk_ESS | Tail_ESS |
| --- | --- | --- | --- | --- | --- | --- | --- |
| Intercept | -1.37 | 0.1 | -1.57 | -1.18 | 1.00 | 22232 | 18238 |
| SubtypeEMSS | -3.48 | 0.4 | -4.34 | -2.77 | 1.00 | 34160 | 16570 |
| Subtypefood | -2.14 | 0.22 | -2.59 | -1.71 | 1.00 | 33911 | 17571 |
| SubtypePS | -4.91 | 0.78 | -6.68 | -3.64 | 1.00 | 29630 | 13164 |
| SubtypePSQT | -25.75 | 30.73 | -98.89 | -5.66 | 1.00 | 4423 | 2697 |
| Subtypetool | -1.69 | 0.19 | -2.07 | -1.33 | 1.00 | 33796 | 17815 |
| sd(Intercept) ss_ID | 0.32 | 0.14 | 0.04 | 0.58 | 1.00 | 4797 | 7081 |
| WAIC | 1464.32 | 70.71 |  |  |  |  |  |

# Who

| Variables | Estimate | Std..Error | Lower.95. | Upper.95. | Rhat | Bulk_ESS | Tail_ESS |
| --- | --- | --- | --- | --- | --- | --- | --- |
| Intercept | -4.66 | 0.35 | -5.41 | -4.02 | 1.00 | 11551 | 11978 |
| SubtypeEMSS | 2.69 | 0.36 | 2.03 | 3.44 | 1.00 | 12050 | 13198 |
| Subtypefood | -0.63 | 0.59 | -1.83 | 0.48 | 1.00 | 18194 | 17418 |
| SubtypePS | 1.16 | 0.4 | 0.42 | 1.97 | 1.00 | 12677 | 14499 |
| SubtypePSQT | 0.31 | 0.46 | -0.58 | 1.20 | 1.00 | 14463 | 15295 |
| Subtypetool | -0.12 | 0.51 | -1.12 | 0.86 | 1.00 | 15813 | 17122 |
| sd(Intercept) ss_ID | 0.54 | 0.15 | 0.23 | 0.84 | 1.00 | 6837 | 7789 |
| WAIC | 1239.43 | 74.4 |  |  |  |  |  |

# Why

| Variables | Estimate | Std..Error | Lower.95. | Upper.95. | Rhat | Bulk_ESS | Tail_ESS |
| --- | --- | --- | --- | --- | --- | --- | --- |
| Intercept | -3.32 | 0.2 | -3.73 | -2.95 | 1.00 | 11780 | 13788 |
| SubtypeEMSS | 0.34 | 0.24 | -0.12 | 0.81 | 1.00 | 15038 | 17167 |
| Subtypefood | -1.27 | 0.37 | -2.03 | -0.57 | 1.00 | 21019 | 18126 |
| SubtypePS | -0.64 | 0.3 | -1.25 | -0.07 | 1.00 | 18207 | 17673 |
| SubtypePSQT | 1.09 | 0.21 | 0.68 | 1.51 | 1.00 | 13376 | 15377 |
| Subtypetool | 1.23 | 0.21 | 0.82 | 1.65 | 1.00 | 13537 | 16603 |
| sd(Intercept) ss_ID | 0.62 | 0.1 | 0.44 | 0.83 | 1.00 | 10273 | 14747 |
| WAIC | 2017.66 | 85.04 |  |  |  |  |  |
