# Supplementary material for: Abstract and concrete concepts in conversation
Source: Sci Rep. 2022 Oct 20;12:17572. doi: 10.1038/s41598-022-20785-5 (PMC9584910; doi:10.1038/s41598-022-20785-5)
Supplement: Supplementary file 4 — Supplementary Information 4. [file 41598_2022_20785_MOESM4_ESM.docx]

**Sentence Coding Guide**

**Conversational domain**

*Number of Questions*

The number of questions produced by participants for the target sentence.

*Type of Questions*

Participant asks Why, How, Where, What, When, and Who questions in simple and articulated structure (e.g., “Where?”, Why?”, “When?”, “How do you feel?”, “What reason?”, “For who/whom?”)

*Uncertain Expressions*

Participants express doubt, uncertainty (e.g., mmh?, and so?), and requests for clarification (What do you mean?).

*Point of View*

Perspective-taking by participants. It refers only to a person/agent and does not include objects/entities.

- 1st person perspective: Participant talks about themselves (e.g., I have committed a sin: I have made many; I thought about the destiny: I should too).
- 2nd person perspective: Participant addresses the other’s viewpoint (e.g., I have committed a sin: Don’t worry!, How do you feel?, Don’t give up).
- 3rd person perspective: Participant talks about s/he viewpoint (e.g., I have committed a sin: My friend made many; How did she react?).
- 1st & 2nd person (i.e., interactive point of view): Participant assumes an interpersonal perspective (e.g., I avoid a conflict: I am happy for you, so next time you avoid making the same mistake).

*General Statement*
Participant is expressing a statement, a general opinion, a common knowledge (e.g., I felt the calm: It's a beautiful feeling; I understood the reason: It’s fundamental; I did a kindness: Empathy is a human feeling).

*Turn-taking*

Participant is trying to establish a conversation, for example asking questions or using expressions signaling the willingness of knowing more details. Turn includes expressions e.g., and?, Tell me, I listen to you, Do you want to talk about it? No turn was coded when participants expressed a simple, short replay (e.g., ok, thank you, good,) or a general statement (e.g., Dream is important).

*Agreement/Disagreement*
Level of agreement/disagreement emerged between the speakers. Agreement: Participant explicitly agrees with the speaker (e.g., Me too, I am with you, You are right, Perfect). Disagreement: Participant explicitly disagrees with the speaker (e.g., I don’t see any reason). No score was coded when there is neither explicit agreement nor disagreement with speakers (e.g., it’s beautiful, ok, good, where?).

*Repetitions of Target word*
The number of repetitions of target words produced for each sentence (e.g., I saw a cow – Where you saw the cow?)

*Number of Evoked Context*
The total amount of context evocated by each sentence (e.g., two contexts = I have demarked the area- Beware of thieves/Is it a new project?)

*Number of Words*
The numbers of words produced by participants in each response (e.g., I am happy for you = 5).

**Sensorimotor grounding**

*Perceptual evaluations*

Participant refers to five sensory modalities.
- Vision: a visual property of the referent (e.g., banana-yellow, zucchini-cubes, room, clean).
- Touch: a tactile property of the referent (e.g., table- smooth).
- Hearing: an auditory property of the referent (e.g., hammer-noise).
- Smell: an olfactory property of the referent (e.g., cake-fragrance).
- Taste: a gustatory property of the referent (e.g., strawberry-good/bitter/I like it).

*Materials/Components*
Participant refers to a material/component comprising the concept (e.g., banana-potassium, bottle-glass, mushroom-toxic, lamp-light/dark).

**Inner grounding**

*Interoception*
Participant refers to inner bodily states (e.g., I have a heartbeat, I am hungry/thirst, I have a stomachache)

*Emotion*
Participant refers to an emotional/ affective state (e.g., I am not vengeful, I am happy, I was ashamed, How do you feel?, I am glad, I hope it works).

*Metacognition*
Participant refers to their own cognitive processes and mental operations (e.g., I estimated the acceleration: I am not able to study physics/ I am good at memorizing; I had a sympathy: I remember when I thought to him).

*Belief/Intentions*
Participant is expressing a belief explicitly (e.g., I think that…, In my opinion…, I believe, I need it, I should do that) or implicitly (e.g., You don’t do that, It was necessary).

*Introspection*
Participant examines their memories (e.g., I made a cake: I remember when my grandmother prepared my birthday cake; conflict: I have long been in conflict with my physical aspect), feelings (I felt ashamed: I often feel ashamed when I speak in public), and experiential states that the speaker is aware of (I overcome the fear: I am sure my fear of insects is exaggerated, but I can’t control it).

**Thematic relations**

*Space*Participant refers to the space or location in which the concept may be found or occurs (e.g., lion: at zoo/in Africa)

*Time*Participant refers to the time when an entity or event occurs (e.g., childhood), and the time period associated with a concept (e.g., I decorated the pumpkin: It is Halloween; I picked up a flower: the spring is coming).

*Events*
Participant mentions events or situations associated with a concept (e.g., I eat a banana: For breakfast?; I thought about money: there is a crisis/I spent a lot of money during my holiday; I am sad: Did you break up?/ I am sorry that you have lost that opportunity).

*Concrete Action****s***
Participant mentions an event involving an agent in action with material entities (e.g., I bought a pencil: now you can draw).

*Abstract Actions*
Participant mentions an event involving an agent in action with immaterial entities (e.g., I solved the enigma - Did you share it?), including advice and exhortations (e.g., I felt anxiety - Don’t give up, Don’t think about it).

**Other**

*Associations*
Participant mentions an object or word associated with the entity tending to co-occur in linguistic or real-world contexts (e.g., I believe in salvation- of the spirit; I dream heaven- it better the hell)

*Subordinates*
Participant adds a specification of a noun, object, or entity (e.g., I bought a bottle - of wine, I make a cake – With chocolate?)

*Non Perceptual Evaluations*
Participant evaluates functional proprieties of an object (e.g., good, useful, nice, old, broken), makes a value judgment on a person (e.g., great! congratulations!), or about action and behavior (e.g., you did well, It was correct/serious/exact).
